# Dynamical Variations of the Global COVID‐19 Pandemic Based on a SEICR Disease Model: A New Approach of Yi Hua Jie Mu

**DOI:** 10.1029/2021GH000455

**Published:** 2021-08-01

**Authors:** Xia Wang, Gang Yin, Zengyun Hu, Daihai He, Qianqian Cui, Xiaomei Feng, Zhidong Teng, Qi Hu, Jiansen Li, Qiming Zhou

**Affiliations:** ^1^ School of Mathematics and Information Science Shaanxi Normal University Xian China; ^2^ College of Resource and Environment Science Xinjiang University Urumqi China; ^3^ State Key Laboratory of desert and Oasis Ecology Xinjiang Institute of Ecology and Geography Chinese Academy of Sciences Urumqi China; ^4^ Research Center for Ecology and Environment of Central Asia Chinese Academy of Sciences Urumqi China; ^5^ University of Chinese Academy of Sciences Beijing China; ^6^ Department of Applied Mathematics Hong Kong Polytechnic University Hong Kong SAR China; ^7^ School of Mathematics and Statistics Ningxia University Yinchuan China; ^8^ School of Mathematics and Informational Technology Yuncheng University Yuncheng China; ^9^ College of Mathematics and System Sciences Xinjiang University Urumqi China; ^10^ School of Natural Resources and Department of Earth and Atmospheric Sciences University of Nebraska Lincoln Lincoln NE USA; ^11^ Guangdong Provincial Center for Disease Control and Prevention Guangzhou China; ^12^ Department of Geography Hong Kong Baptist University Hong Kong China

**Keywords:** COVID‐19 pandemic, Koppen‐Geiger climate classification, periodic variation, scenario analysis

## Abstract

The ongoing coronavirus disease 2019 (COVID‐19) pandemic has caused more than 150 million cases of infection to date and poses a serious threat to global public health. In this study, global COVID‐19 data were used to examine the dynamical variations from the perspectives of immunity and contact of 84 countries across the five climate regions: tropical, arid, temperate, and cold. A new approach named Yi Hua Jie Mu is proposed to obtain the transmission rates based on the COVID‐19 data between the countries with the same climate region over the Northern Hemisphere and Southern Hemisphere. Our results suggest that the COVID‐19 pandemic will persist over a long period of time or enter into regular circulation in multiple periods of 1–2 years. Moreover, based on the simulated results by the COVID‐19 data, it is found that the temperate and cold climate regions have higher infection rates than the tropical and arid climate regions, which indicates that climate may modulate the transmission of COVID‐19. The role of the climate on the COVID‐19 variations should be concluded with more data and more cautions. The non‐pharmaceutical interventions still play the key role in controlling and prevention this global pandemic.

## Key Points

- A new approached is proposed to predict the future COVID‐19 variations rather than relying on information on other corona viruses
- COVID‐19 pandemic will persist in multiple periods of 1–2 years
- The temperate and cold climate regions have higher infection rates than the tropical and arid climate regions

## 1 Introduction

Rapidly spreading and ravaging the world, severe acute respiratory syndrome‐coronavirus 2 (SARS‐CoV‐2) has caused the coronavirus disease 2019 (COVID‐19) pandemic through human‐to‐human transmission (Armitage & Nellums, 2020; Chinazzi et al., 2020), resulting in more than 158,000,000 total confirmed cases and more than 3,000,000 deaths in more than 200 countries/regions as of May 11, 2021 (WHO, https://covid19.who.int/). This global pandemic has serious impacts on public health and on social and economic development (Baker et al., 2020; Zerhouni et al., 2020). A great number of measures have been quickly adopted to reduce the transmission and to mitigate the impact of the pandemic (Cohen & Corey, 2020; Hsiang et al., 2020; Thorp, 2020). The effective measures and strategies employed in China provided a useful example to other countries in preventing and curing COVID‐19 (Guan et al., 2020; Kraemer et al., 2020; Wu & McGoogan, 2020; F. Zhou et al., 2020).

However, there is neither a specific drug nor vaccine treatment for COVID‐19 because typically months to years are needed to develop and test such therapeutics (Ferretti et al., 2020; Tian et al., 2020). Therefore, non‐pharmaceutical interventions have been widely used by all countries as the only immediate means of curbing SARS‐CoV‐2 transmission, for example, physical (social) distancing, closing schools and workplaces, limiting the sizes of gatherings, wearing face masks and eye protection, and quarantine (Ali et al., 2020; Chu et al., 2020; Cui et al., 2020; Giordano et al., 2020; Hu et al., 2020; Chinazzi et al., 2020; Parmet & Sinha, 2020; Ruktanonchai et al., 2020; Sjodin et al., 2020). Physical distancing as implemented in China during the outbreak has been able to control COVID‐19 (Zhang et al., 2020), and the national emergency response has delayed the growth and limited the size of the COVID‐19 spread in China, averting hundreds of thousands of cases (Prem et al., 2020; Tian et al., 2020). Restrictive physical distancing measures combined with widespread testing and contact tracing could end the ongoing COVID‐19 pandemic (Britton et al., 2020; Giordano et al., 2020; Hao et al., 2020; Lai et al., 2020).

To employ the correct measures at the right time in controlling the COVID‐19 pandemic, it is of crucial importance to accurately understand the routes and timings of transmission, especially accurate prediction of COVID‐19 variations in the future (Kissler et al., 2020). Mathematical models can not only probe the complexity of infectious disease dynamics (e.g., period, bifurcation, and chaos), but can also elucidate the mechanisms of transmission and indicate new approaches for prevention and control strategies (Heesterbeek et al., 2015). Assuming that the COVID‐19 pandemic adapts to similar climate scenarios based on known coronavirus biology, it will exhibit seasonal variations and become a seasonal epidemic according to the results of a climate‐dependent epidemic model (Baker et al., 2020). Based on a SEIRS epidemic model, it was proposed in a recent work that COVID‐19 can exist at any time of year, and it will likely enter into regular circulation if immunity to SARS‐CoV‐2 is not permanent (Kissler et al., 2020). However, they only used less than five years data of betacoronaviruses HCoV‐OC43 and HCoV‐HKU1 to predict COVID‐19 variations which is a serious limitation based on the transmission characteristics of known coronavirus strains (Baker et al., 2020; Kissler et al., 2020).

It is well known that climate changes have significant impacts on large of human diseases which are concluded using numerous long term disease data sets and climate data sets, such as the impacts of temperature and specific humidity on the human influenza infections (W. Liu et al., 2019; Shaman et al., 2010; Tamerius et al., 2013), and the positive influence of low temperature and low relative humidity on the coronaviruses (Aboubakr et al., 2020; Sundell et al., 2016; Yang & Marr, 2011).

In terms of the COVID‐19, the role of climate in COVID‐19 mitigation strategies is still a dispute topic (OReilly et al., 2020). Although some literatures (Araujo & Naimi, 2020; J. Liu et al., 2020; Sajadi et al., 2020) explore the impacts of climate factors (e.g., temperature and specific humidity) on the COVID‐19 variations and suggest that SARS‐CoV‐2 is less transmissible in hot and humid climates, there is no sufficient evidence supporting that large numbers of COVID‐19 cases are associated with cold and dry climates due to only not less than two years data (Baker et al., 2020; OReilly et al., 2020; Prata et al., 2020).

Environment changes (e.g., climate changes) affect the outbreak and transmission of many diseases directly or indirectly (Baker et al., 2021; Tamerius et al., 2013). Specific humidity has been shown to be important for influenza transmission in both laboratory settings and population‐level studies. Therefore, it is important explore the disease transmission or outbreak characteristics in geospatial perspectives.

However, with limited data on the current epidemic, these early stage results are inevitably inconclusive. Furthermore, the relative importance of climate drivers when compared with high population susceptibility during the pandemic stage of an emerging infection such as SARS‐CoV‐2 has not been fully characterized (Baker et al., 2020; Paraskevis et al., 2021). Therefore, any COVID‐19 risk evaluations and predictions based on climate information alone should be interpreted with caution. The role of the climate changes on the COVID‐19 variations will be not explored in this study because of the limited information from the no more than two years’ COVID‐19 transmission.

Projecting the transmission dynamics of the global COVID‐19 pandemic is very important and urgent in order to employ the correct strategies and measures to control the outbreak of this disease. For the study of the global COVID‐19 pandemic, the following questions must first be addressed (a) What are the differences in the present transmission of COVID‐19 in the different climate regions of various countries? (b) Does a reasonable approach exist to explore the future changes of COVID‐19 in the world, but not as previous studies based on known coronavirus strains? (c) What are the future risks of the global COVID‐19 pandemic?

To address the above questions, this study aimed to (a) evaluate and predict the transmission dynamics of the COVID‐19 pandemic over different climate regions, (b) to propose an innovated approach to investigate the future dynamical behaviors rather than relying on information on other coronaviruses, and (c) to explore the COVID‐19 variations using different strategies in future. These analyses are only interpreted based on the COVID‐19 data objectively.

## 2 Methods

### 2.1 SEICR Model

**Figure 1:**
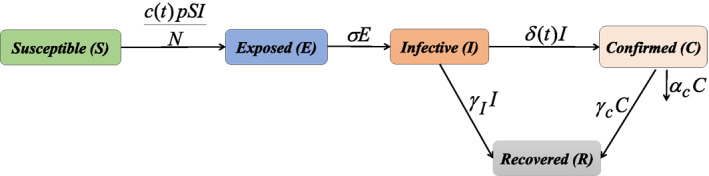
Flowchart of COVID‐19 SEICR epidemic model.

Based on the transmission characteristics of the COVID‐19 pandemic and previous literatures (Cui et al., 2020; Hu et al., 2020), the entire population at time t is divided into five components, that is, susceptible individuals S(t), exposed individuals E(t), infectious individuals I(t), confirmed individuals C(t), and removed individuals R(t). We assume that the confirmed individuals C(t) cannot transmit among the population because they will be quarantined if they are confirmed. The COVID‐19 disease is transmitted from S(t) to E(t) by the contact behaviors and the transmission characteristic of the SARS‐CoV‐2 composed a standard incidence rate. The exposed individuals E(t) transitions to the infectious individuals I(t) in a rate. Part of I(t) becomes the confirmed individuals C(t) by the COVID‐19 detection, and the other I(t) transitions to the removed individuals R(t) in a recovery rate. The confirmed individuals C(t) becomes death partly and the residual will be recovered as the removed individuals R(t). The details of the disease transmission among the different individuals are well illustrated by the flowchart figure (Figure 1).

According to the above analysis, the corresponding SEICR disease model can be described by the following system of ordinary differential equations:

(2.1) $$\left\{\begin{array}{c} S^{\prime }=-\frac{c\left(t\right)pSI}{N},\;\\ E^{\prime }=\frac{c\left(t\right)pSI}{N}-\sigma E,\;\\ I^{\prime }=\sigma E-\left(\delta \left(t\right)+\gamma_{I}\right)I,\;\\ C^{\prime }=\delta \left(t\right)I-\left(\alpha_{c}+\gamma_{c}\right)C,\;\\ R^{\prime }=\gamma_{I}I+\gamma_{c}C,\; \end{array}\right.$$

where the contact rate function is

(2.2) $$c\left(t\right)=\left\{\begin{array}{c} c_{0},\;t\leq t_{c}\\ \left(c_{0}-c_{f}\right)e^{-r_{b}\left(t-t_{c}\right)}+c_{f},\;t>t_{c} \end{array}\right.$$

and the detection rate function is

(2.3) $$\frac{1}{\delta \left(t\right)}=\left\{\begin{array}{c} \frac{1}{\delta_{0}},\;t\leq t_{c}\\ \left(\frac{1}{\delta_{0}}-\frac{1}{\delta_{f}}\right)e^{-r_{d}\left(t-t_{c}\right)}+\frac{1}{\delta_{f}}.\;t>t_{c}. \end{array}\right.$$

c(t) is the contract rate which is determined by many factors, such as population density, total population and traffic types. $c_{0}$ is the contact rate and $\delta_{0}$ is the detection rate at the early disease transmission period $t_{c}$. $c_{f}$ is the minimum contact rate under the current control strategies. $r_{b}$ denotes the contact rate modeled as an exponentially decreasing rate, which assumes that the contact times are decreasing with the implementation of intervention.

δ(t) is the detection rate of the COVID‐19 disease that is mainly resulted by the level of the public health system, the medical resources and the gross domestic product (GDP). $\delta_{f}$ is the maximum detection rate under the current control strategies, and each country has its own maximum detection rate value. $r_{d}$ denotes the exponentially decreasing rate of the testing period. Considering that the contact rate and detection rate will gradually decrease or increase with the gradual strengthening of control measures, and finally reach the minimum contact rate or maximum detection rate, we use the above function form as shown in the literature (Tang et al., 2020).

Parameter p is the transmission rate of COVID‐19, which depends on the SARS‐CoV‐2 virus. σ is the transition rate from exposed individuals E(t) to infectious individuals I(t). $\gamma_{I}$ and $\gamma_{c}$ are the recovery rates of I(t) and C(t), respectively. $\alpha_{c}$ is the death rate of C(t). During the incubation period of 14 days (Lauer et al., 2020), for some COVID‐19 cases, it is difficult to develop symptoms. Therefore, $t_{c}=14\;\text{days}$ is set as the key time period in which the prevention and control measurements are not employed in different countries over the world. Parameters except σ and $t_{c}$ are estimated by fitting the model to data (cumulatively number of confirmed cases, cumulatively number of recovered cases and cumulatively number of deaths), by the nonlinear least square method as previous study (Cui et al., 2020; Hu et al., 2020). Definitions of the parameters are shown in Table 1.

**Table 1:** Definitions of the Parameters Used in the Model

Parameter	Definition (units)	Value	References
c(t)	The contact rate at time t		Estimated
$c_{0}$	The initial contact rate		Estimated
$c_{f}$	The minimum contact rate		Estimated
$r_{b}$	The exponential decreasing rate of the contact rate		Estimated
$t_{c}$	The time period before control	14	Assumed
p	The probability of transmission per contact		Estimated
δ(t)	The detection rate at time t		Estimated
$\delta_{0}$	The initial detection rate		Estimated
$\delta_{f}$	The maximum detection rate		Estimated
$r_{d}$	The exponential increasing rate of the detection rate		Estimated
σ	The transition rate from E to I	1/5	Tang et al. (2020)
$\gamma_{I}$	The recovery rate of I		Estimated
$\gamma_{c}$	The recovery rate of C		Estimated
$\alpha_{c}$	The death rate of C		Estimated

According to the model (2.1), the controlled reproductive number $R^{*}$ is determined by the parameters of the contact rate c(t), the transmission rate p, the detection rate δ(t), and the recovery rate of $\gamma_{I}$ with the following form:

(2.4) $$R^{*}=\frac{c\left(t\right)p}{\delta \left(t\right)+\gamma_{I}},$$

which indicates the average secondary cases infected by one infected individual in the infectious period.

When $c\left(t\right)=c_{0}$ and $\delta \left(t\right)=\delta_{0}$, the controlled reproductive number $R^{*}$ is the basic reproductive number $R_{0}$

(2.5) $$R_{0}=\frac{c_{0}p}{\delta_{0}+\gamma_{I}}.$$

It should be noted that although the COVID‐19 variations between countries may be caused by different factors, such as different climate factors, population densities, and different responses. In this study, we aim to only employ a reasonable and general model addressing the disease variations to avoid large uncertainties induced by these complex factors.

### 2.2 Climate Classification and Selecting the 85 Countries

The Köppen‐Geiger system classifies climate into five main classes and 30 sub‐types. The classification is based on threshold values and seasonality of monthly air temperature and precipitation. The five climatic regions include tropical, arid, temperate, cold, and polar. This classification is identical to that presented by Köppen in 1936 with three differences. First, temperate (C) and cold (D) climates are distinguished using a 0°C threshold instead of a 3°C threshold. Second, the arid (B) sub‐climates W (desert) and S (steppe) were identified depending on whether 70% of precipitation occurred in summer or winter. Third, the sub‐climates s (dry summer) and w (dry winter) within the C and D climates were made mutually exclusive by assigning s when more precipitation falls in winter than in summer and assigning w otherwise. Note that the tropical (A), temperate (C), cold (D), and polar (E) climates are mutually exclusive but may intersect with the arid (B) class. To account for this, climate type B was given precedence over the other classes. The detailed classification can be found in Table 2 of the Methods section of Beck et al. (2018).

**Figure 2:**
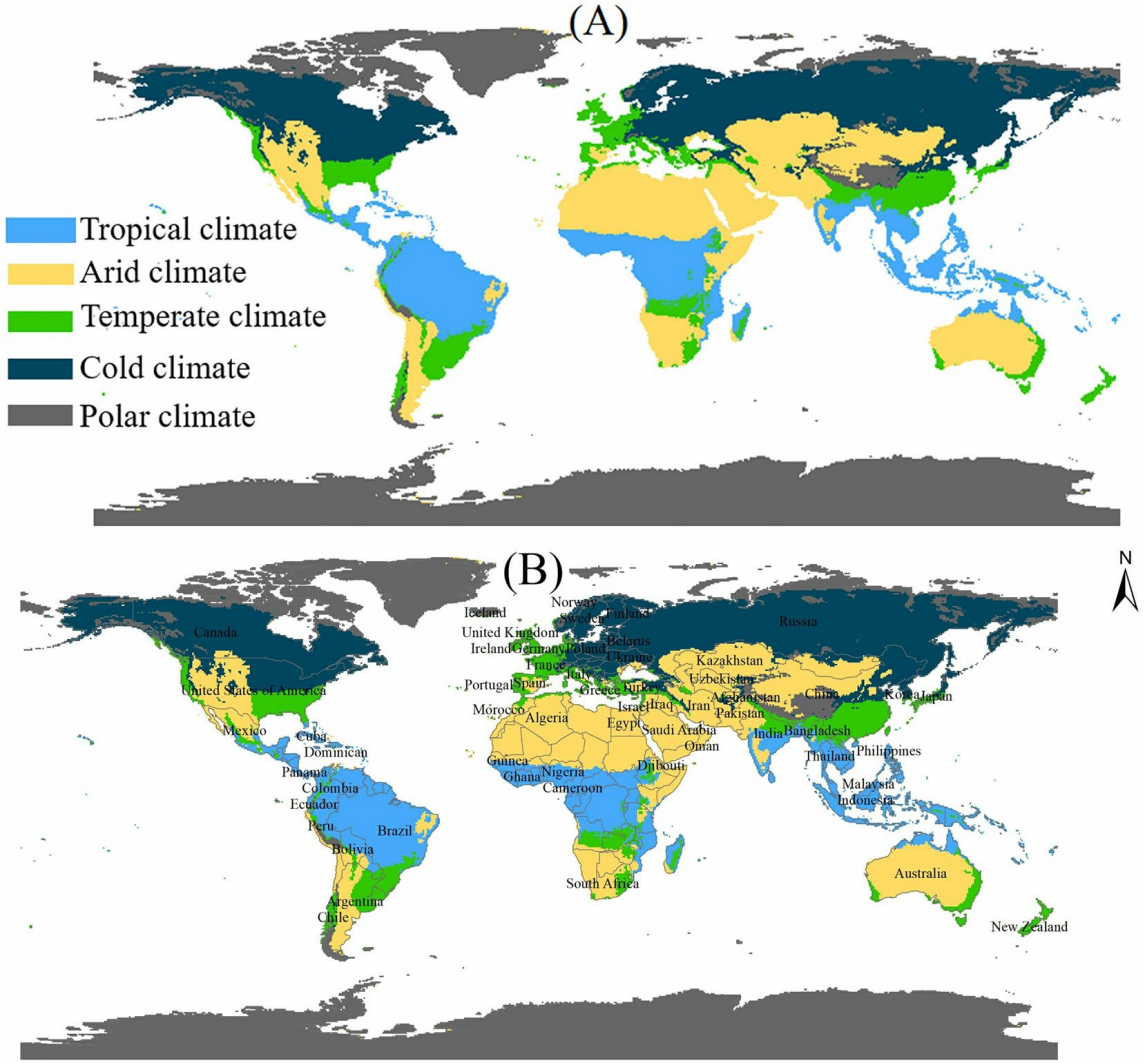
(a) Climate classification result based on the Köppen‐Geiger climate classification maps, where represents the tropical, arid, temperate, cold and polar climate, respectively; (b) 85 countries in the five climate regions.

At April 30, 2020, there are 186 countries reported the COVID‐19 cases with the values from 1 to more than one million. In this study, we only focus on the countries with a number of cumulative confirmed cases larger than 1,000 are considered and are classified based on the Köppen‐Geiger climate classification maps (Figure 2a).

Through April 30, 2020, there were 85 countries with confirmed cases of more than 1,000, which were distributed in the Northern Hemisphere (NH), totaling 78 countries, and in the Southern Hemisphere (SH), totaling seven countries (Figure 2b). In our study, if a country covers more than two climate types, it will be classified in the climate region with the largest area. Then, for different climate regions, there are 17 countries in the tropical region, 27 countries in the arid region, 16 countries in the temperate region, 24 countries in the cold region, and one country in the polar region (Figure 2b and Table S1). Due to only one country in the polar region, this climate region is not considered in the following study, and the simulation and sensitivity analyzes focus on the 84 countries distributed over the four climate regions.

### 2.3 A New Approach of Yi Hua Jie Mu

A new approach is proposed herein to predict the COVID‐19 dynamical behaviors and is based on the following hypotheses.

1. Two seasons are defined, including a warm season (May–October) and a cold season (November–April).
2. In the same season, the same climate regions across the NH and SH have the same transmission rates of the SARS‐CoV‐2 virus. For example, in the warm season, NH and SH have the same transmission rate across the same climate regions.
3. Because the countries in the NH and SH experience opposite seasons during the same time period (e.g., from November 2019 to April 2020 defined the cold season in the NH and the warm season in the SH), for the same climate region, the COVID‐19 transmission of the countries of the NH in the warm season with a fixed infection rate $p^{*}$ computed by the data in the countries of the SH is predicted without using the infection rate obtained by the COVID‐19 data set from the cold season, and vice versa. This new approach is named Yi Hua Jie Mu.
4. $p^{*}$ is established by the COVID‐19 data using the SEICR model. To remove the uncertainties of the $p^{*}$ obtained from the countries in the same climate regions across NH, $p^{*}$ used in the COVID‐19 prediction of the countries in the NH is averaged by the transmission rates of different countries in the SH, and vice versa. For example, for each climate region, the transmission rate of $p^{*}$ used in predicting the future disease variations in the NH have the following form

(2.6) $$p^{*}=\left\{\begin{array}{c} p_{1},\;\text{cold}\;\text{season},\\ p_{2},\;\text{warm}\;\text{season}, \end{array}\right.$$

where $p_{1}$ is averaged from the transmission rates of the countries in the NH in the cold season by the data (if available) from November 2019 to April 2020, and $p_{2}$ is averaged from the transmission rates of the countries in the SH in the warm season by the data from November 2019 to April 2020.

5. Since there is no obvious difference in the climate between the warm and cold seasons in tropical regions, the infection rate used in prediction is stilled obtained by the historical data of the countries in the NH and SH, respectively.
6. When predicting future COVID‐19 transmission, it is assumed that immunity to SARS‐CoV‐2 is not permanent for different scenarios with mR from the recovered individuals to the susceptible individuals again, and $\frac{1}{m}$ is the immune period (in days). The model is as follows

(2.7) $$\left\{\begin{array}{c} S^{\prime }=-\frac{c\left(t\right)pSI}{N}+mR,\;\\ E^{\prime }=\frac{c\left(t\right)pSI}{N}-\sigma E,\;\\ I^{\prime }=\sigma E-\left(\delta \left(t\right)+\gamma_{I}\right)I,\;\\ C^{\prime }=\delta \left(t\right)I-\left(\alpha_{c}+\gamma_{c}\right)C,\;\\ R^{\prime }=\gamma_{I}I+\gamma_{c}C-mR,\; \end{array}\right.$$

In the simulation process, $t_{c}$ is assumed to be 14 days. The length of the time series for each country is defined as $t_{*}$. For the contact rate $c_{0}$ and $c_{f}$, they are certainly and majorly determined by the population number, population density, culture and travel habits which are difficult to obtain the empirical values. Therefore, they are estimated by fitting model to data. To investigate the impact of immunity and contact parameters on the future transmission period of the COVID‐19 pandemic ($t>t_{*}$), several assumptions were made regarding the immune loss rate m and contact rate c. Immune loss rates are $m=0,\;\frac{1}{365}$, and $\frac{2}{365}$, which indicate permanent immunity, one year immunity, and half‐year immunity, respectively. The corresponding contact rates are $c=c_{f},\;1.2c_{f}$, and $c_{0}$.

Then, there are nine scenarios for the above immune loss rates and contact rates:

Scenario 1 (S1): m=0, $c=c_{f}$;.
Scenario 2 (S2): $m=\frac{1}{365}$, $c=c_{f}$;.
Scenario 3 (S3): $m=\frac{2}{365}$, $c=c_{f}$;.
Scenario 4 (S4): m=0, $c=1.2c_{f}$;.
Scenario 5 (S5): $m=\frac{1}{365}$, $c=1.2c_{f}$;.
Scenario 6 (S6): $m=\frac{2}{365}$, $c=1.2c_{f}$;.
Scenario 7 (S7): m=0, $c=c_{0}$;.
Scenario 8 (S8): $m=\frac{1}{365}$, $c=c_{0}$;.
Scenario 9 (S9): $m=\frac{2}{365}$, $c=c_{0}$.

### 2.4 Estimating the Parameters and Fitting the Model

The parameters of model (2.1) and model (2.6) are estimated by the nonlinear least square method by fitting model to the number of cumulative confirmed cases($Y_{c}\left(t\right)$), number of recovered cases($Y_{r}\left(t\right)$), and number of death cases($Y_{d}\left(t\right)$). The objective function for our model (2.1) is

$$L\left(\theta \right)=\Sigma_{i=1}^{T}\left[{\left(C_{c}\left(t\right)-Y_{c}\left(t\right)\right)}^{2}+{\left(C_{d}\left(t\right)-Y_{d}\left(t\right)\right)}^{2}+{\left(C_{d}\left(t\right)-Y_{r}\left(t\right)\right)}^{2}\right]$$

where $dC_{c}\left(t\right)/dt=\delta \left(t\right)I,dC_{d}\left(t\right)/dt=\alpha_{c}C$ and $dC_{r}\left(t\right)/dt=\gamma_{c}C$. T is the length of the data and $\theta =\left(E_{0},\;I_{0},\;c_{0},\;\delta_{0},\;\alpha_{c},\;\gamma_{I},\;\gamma_{c},\;c_{f},\;r_{b},\;\delta_{f},\;r_{d},\;p\right)$.

After obtained the estimated parameters, the simulated COVID‐19 data and the predicted COVID‐19 data will be computed by the model (2.1) and model (2.7) using the estimated parameters. The model performance (or the simulation accuracy) is quantitatively measured by the correlation coefficient (CC), the relative bias (RB) and the distance between indices of simulation and observation (DISO) as previous studies (Cui et al., 2020; Hu et al., 2020). DISO is developed to describe the overall performances of the simulated models against the observed field quantitatively (Hu et al., 2019; Q. Zhou et al., 2021). The values of the estimated parameters, CC, RB, and DISO of the 84 countries are provided in Table S2.

### 2.5 Framework of This Study

**Figure 3:**
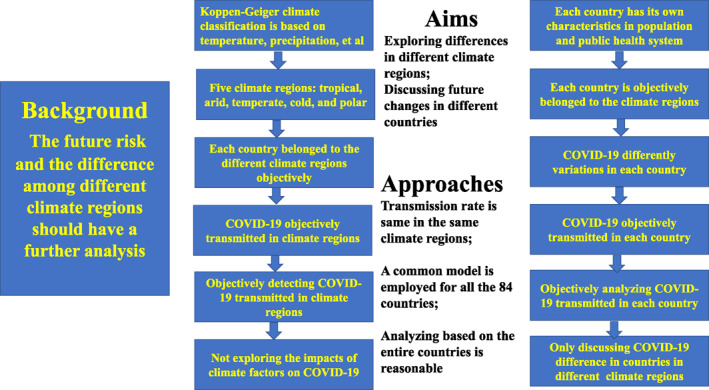
Framework of this study.

From the above analysis, three issues should be emphasized and clarified again. The first issue is that the role of the climate factors on the COVID‐19 variations are excluded in this study. The second issue is that a general disease model is established for all the 85 countries across the five different climate regions, and the COVID‐19 variations will be analyzed and discussed according to the general model and the COVID‐19 data objectively. The last issue is that the general model cannot include all the factors (e.g., GDP per capita and population density) impacting the COVID‐19 variations. In fact, the detection capacity is mainly determined by the level of the public health system which is largely impacted by the GDP per capita. The contact rate directly reflects the population density. In our model, the detection rate and contact rate are all included. With these issues in mind, the framework of this study is provided in Figure 3 which can help us have a well understanding of the design and structure of this study.

## 3 Data Availability

In this study, the global COVID‐19 pandemic data of 84 countries from the date of the first cases of every country to April 30, 2020 is derived from an R package with real‐time data (https://github.com/GuangchuangYu/nCov2019). The COVID‐19 pandemic data include the number of cumulative confirmed cases, number of recovered cases, and number of death cases. The reason why we chose the data up to April 30, 2020 is that the data set of the early stage of the COVID‐19 transmission has the inherent and the initial characteristics and can avoid many other factors controlled by human activities. The corresponding parameters of the SEICR model established by that period can reflect the initial characteristics.

For each country, the population number is from the 2018 World Health Organization (WHO) data and is considered to be the total population in the simulation and prediction processes. The global shape data were downloaded from https://gadm.org. Global climate is classified into four regions: tropical, arid, temperate and cold, which is based on the latest Köppen‐Geiger climate classification maps at 1‐km resolution (Beck et al., 2018).

## 4 Results

In this section, the simulation results of the COVID‐19 variations and the estimated parameters in Table 2 are first provided. Then, we predict the dynamic variations of the COVID‐19 pandemic in the 84 countries over different climate regions at nine scenarios with the changes of contact rates and immunity rates. The predict period is from the beginning date of the COVID‐19 to the future five years.

**Table 2:** Parameter Values Obtained From the Simulation, Including Contact Rate at Early Transmission Period, $c_{0}$, Minimum Contact Rate $c_{f}$, Transmission Rate p, Basic Reproductive Number $R_{0}^{*}$, and Controlled Reproductive Number $R_{f}^{*}$, Which are Averaged From the Parameter Values of COVID‐19 Data From the 84 Countries Studied

Climate regions	$c_{0}$	$c_{f}$	p	$R_{0}^{*}$	$R_{f}^{*}$
Tropical	13.02	8.01	0.065	4.12	0.83
Arid	11.92	6.99	0.069	4.44	0.81
Temperate	12.02	6.59	0.081	5.33	0.76
Cold	9.62	5.18	0.080	4.17	0.67

### 4.1 Dynamical Variations of the COVID‐19 Pandemic Before May 2020

COVID‐19 was assessed as a pandemic on March 11, 2020 by the WHO with 120,957 cases and 4,390 deaths, and the number of the global cumulative confirmed cases increased to more than 1 million in only 23 days by April 3, 2020. With such rapid transmission, the number of the global cumulative confirmed cases reached more than 2, 4, 6, 8, and 10 million in 24, 12, 21, 16, and 13 days, respectively, which were first reported on April 27, May 9, May 30, June 15, and June 28, 2020, respectively (Figure S1). For the spatial distributions, the United States of America (America), Brazil, India, and Russia contributed to large parts of the global COVID‐19 cases (Figures S1c–S1f). The details of the spatial transmission are obtained in Supporting Information. The climate classification results and the selected 84 countries are displayed in Figure 1, which are identified by the Köppen‐Geiger climate classification maps and the number of the cumulative confirmed cases.

For the simulation, the model (2.1) in this work captured the COVID‐19 variations of the cumulative confirmed cases, cumulative recovered cases, and cumulative deaths for the 84 countries distributed over different climate regions (Figures S2–S6). The CC values between the observed total cumulative confirmed cases and the simulated total cumulative confirmed cases are nearly to one. The RB values are smaller than 0.1. And the corresponding comprehensive performances of the model (2.1) are well evaluated with the DISO values nearly to one (Table S2).

For tropical regions, the COVID‐19 variations of the typical countries of Bolivia, Brazil, Colombia, India, Peru, Philippines, and Singapore are simulated by model (2.1). The cumulative confirmed cases, cumulative recovered cases, and cumulative deaths of Colombia, India, and The Philippines are captured with high accuracy (Figure S2). The variations of cumulative confirmed cases and cumulative deaths of Bolivia and Peru in the SH are well captured.

For typical countries in arid regions, the simulated time series are consistent with the variations of the cumulative confirmed cases, cumulative recovered cases, and cumulative deaths (Figure S3), especially for Chile and Egypt. Moreover, the model has high simulation ability for the countries with confirmed cases larger than 100,000, such as Spain, Turkey, and America. For Mexico and South Africa, the recovered cases are not well captured, which is mainly caused by the quality of the recovered data. The COVID‐19 pandemic variations are well simulated in temperate, cold, and polar regions (Figures S4–S6), such as France, Germany, Italy, and Japan in temperate regions (Figures S4) and Canada, Russia, and South Korea in cold regions (Figure S5). The COVID‐19 pandemic variations of the other countries over the four climate regions are also well simulated (see Figures S2–S6). For most countries, the CC values are larger than 0.9, and the RB values are smaller than 10%.

**Figure 4:**
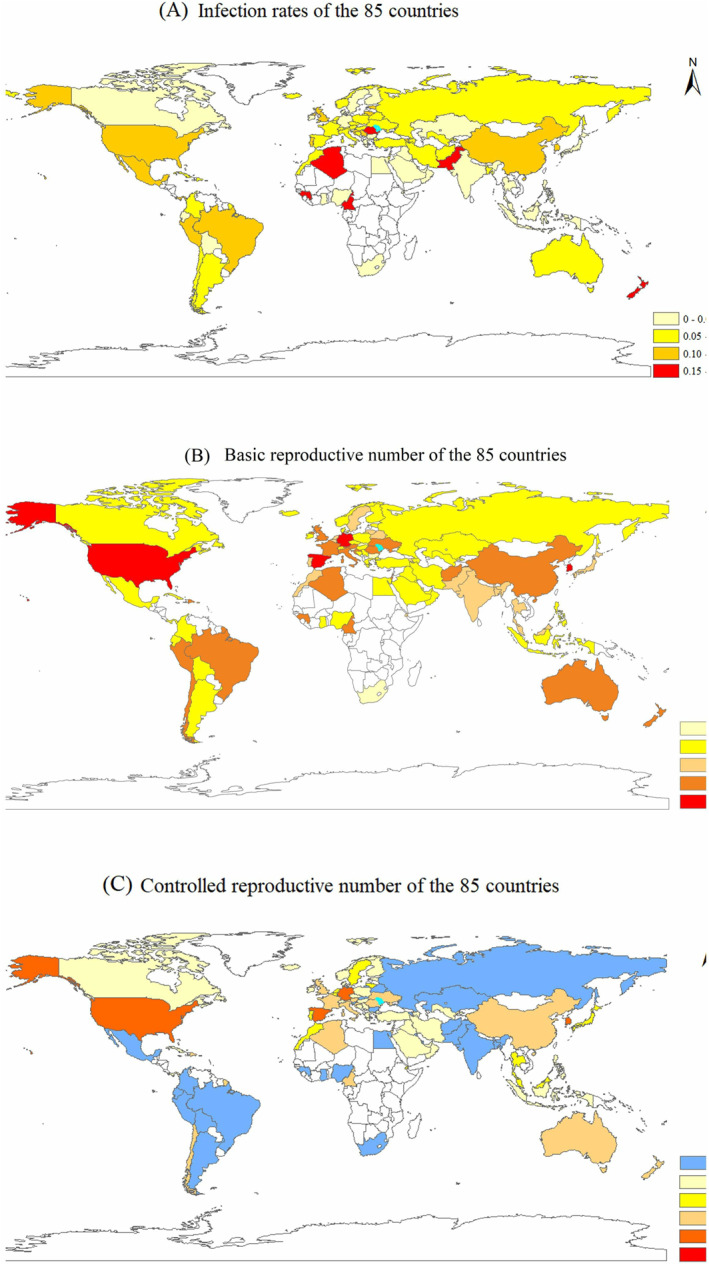
(a) Distributions of the transmission rate p, (b) the basic reproductive number $R_{0}^{*}$, and (c) the controlled reproductive number $R_{f}^{*}$ of the 85 countries.

**Figure 5:**
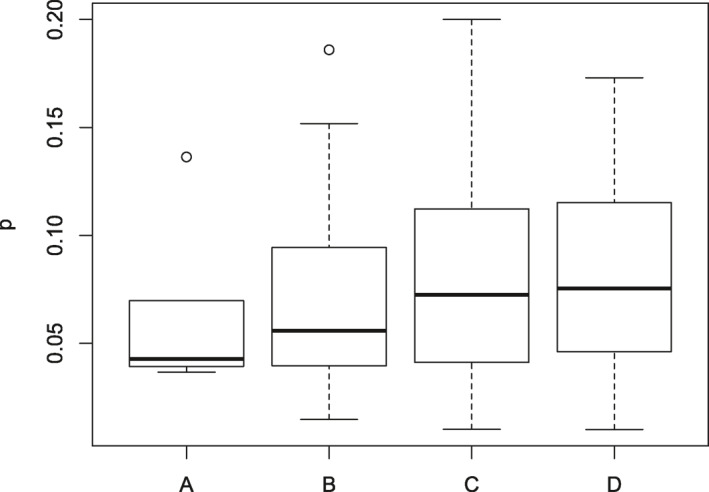
Boxplot of the transmission rates of the 84 countries over four climate regions, where A, B, C, and D represent tropical, arid, temperate, and cold climate regions.

The spatial distributions of the corresponding key parameters of the 84 countries are displayed in Figures 4, S7, and S8. Among the 84 countries, six countries have the transmission rates p larger than 0.15, such as Cameroon, Algeria, and Pakistan, followed by 15 countries with an infection rate between 0.1 and 0.15 (i.e., Brazil, Peru, China, and America in Figure 4a). For the basic reproductive number $R_{0}^{*}$, Spain, Germany, South Korea, Spain, and America have the values larger than 10 (Figure 5b), which explains the large number of confirmed cases in these countries (Figures 1a and 1b). Under the current control strategies, the controlled basic reproductive number $R_{f}^{*}$ decreased to below the disease transmission threshold value $R_{0}^{*}=1$ in approximately 71% of the countries (Figure 5c). The spatial distributions of the contact rates $c_{0}$ at early transmission period and the minimum contact rates $c_{f}$ in Figures S7 and S8 illustrate the spatial distributions of $R_{0}^{*}$ and $R_{f}^{*}$, respectively (Figures 4b and 4c, respectively).

In addition, the averaged parameter values of the 84 countries over the four climate regions using the COVID‐19 data before May 1, 2020 were explored (Table 2). The table shows that the contact rate at the early transmission period, $c_{0}$, and the minimum contact rate $c_{f}$ increased from a cold climate to tropical climate with the values ranging from 9.62 to 13.02 and from 5.18 to 8.01, respectively. The transmission rates p in cold and temperate climate regions with the respective values of 0.08 and 0.081 are larger than those in the arid, and tropical climate regions, that is, 0.069, and 0.065, respectively. Moreover, the boxplot of the transmission rates of the 84 countries over the four climate regions also confirmed that the transmission rates in cold and temperate are larger than the values in the arid and tropical regions (Figure 5). This result indicates that the COVID‐19 pandemic caused by the SARS‐CoV‐2 virus poses a higher risk for transmission in cold and temperate climate regions than in other climate regions. The basic reproductive number $R_{0}^{*}$ of temperate climate regions are the largest compared to those of the other regions at the early transmission period. After some intervention strategies, such as community quarantine, safe social distancing, closing schools and workplaces, limiting the sizes of gathering, and wearing masks, the controlled reproductive number $R_{f}^{*}$ values of the four climate regions are 0.67, 0.76, 0.81, and 0.83 for cold, temperate, arid, and tropical climate regions, respectively.

According to the above analysis, the cumulative confirmed cases of the countries over the different climate regions have the best simulated accuracy compared with the cumulative recovered cases and deaths due to differences in data quality. Therefore, to investigate COVID‐19 pandemic transmission, the focus was on daily new confirmed cases computed from the difference of the cumulative confirmed cases.

### 4.2 Dynamic Variations of the COVID‐19 Pandemic in Different Scenarios

**Figure 6:**
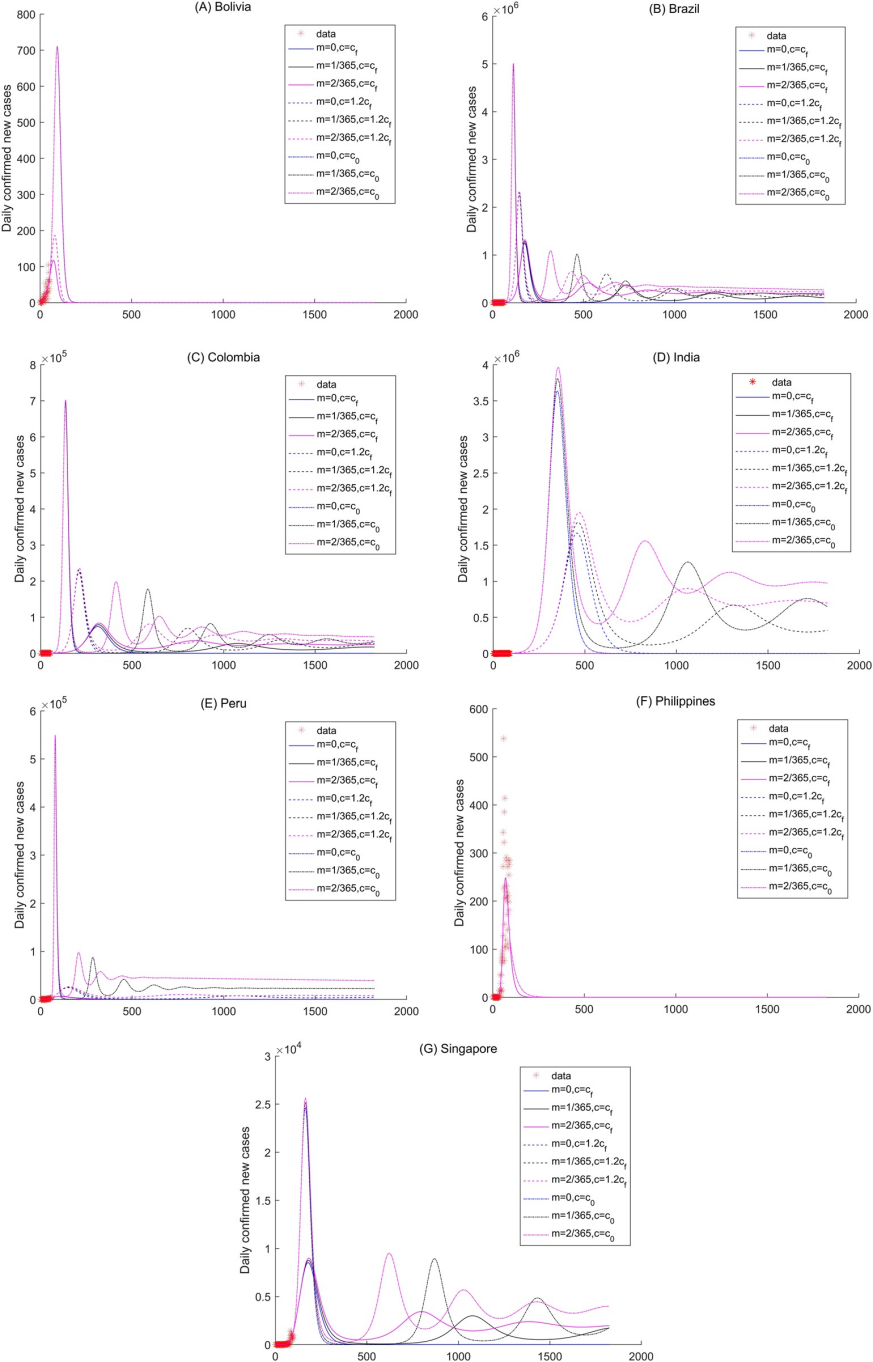
Sensitivity analysis of the daily new confirmed cases of Bolivia, Brazil, Colombia, India, Peru, Philippines, and Singapore in tropical region.

**Figure 7:**
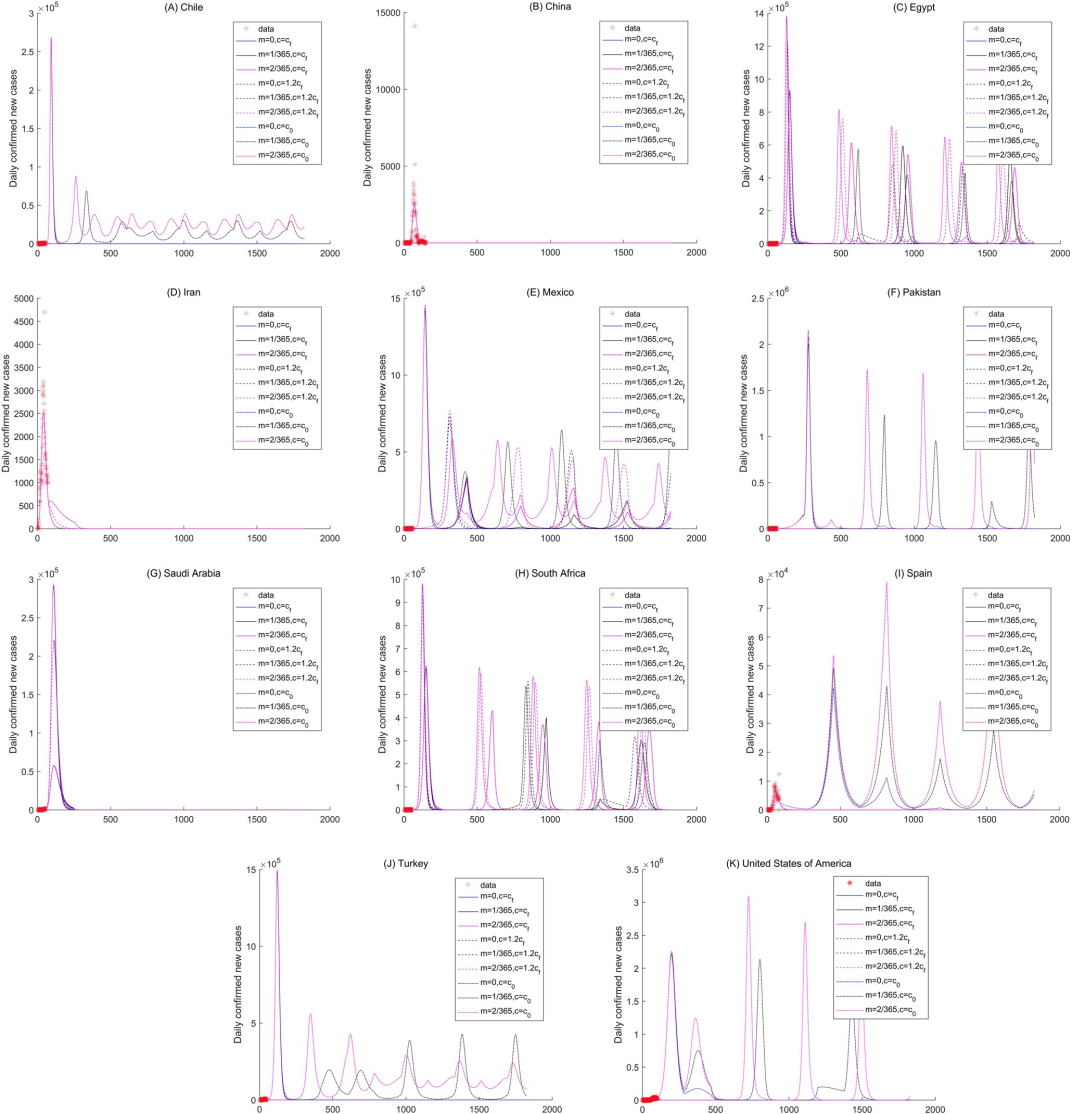
Sensitivity analysis of the daily new confirmed cases of Chile, China, Egypt, Iran, Mexico, Pakistan, Saudi Arabia, South Africa, Spain, Turkey, and United States in arid region.

**Figure 8:**
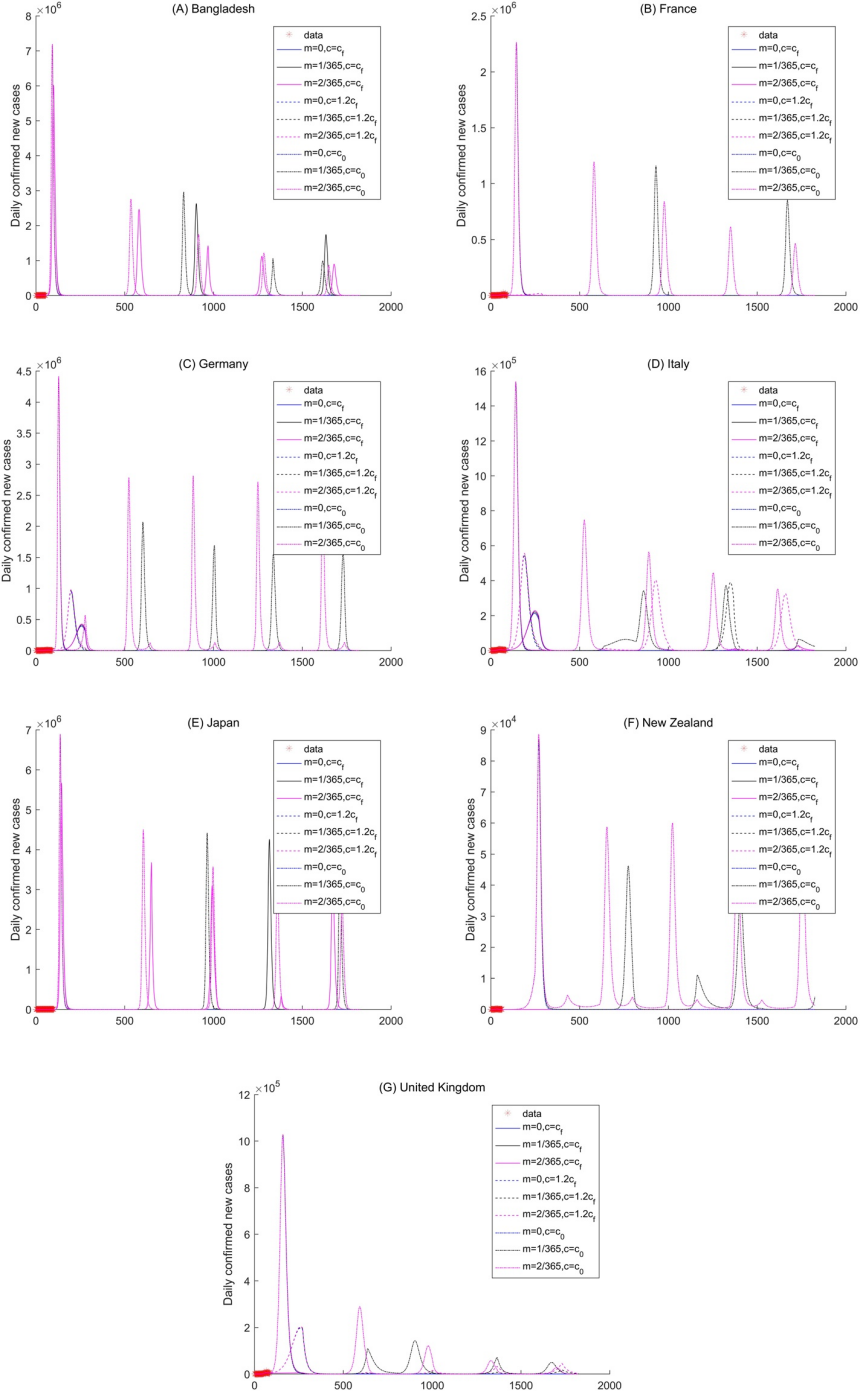
Sensitivity analysis of the daily new confirmed cases of Bangladesh, France, Germany, Italy, Japan, New Zealand, and United Kingdom in temperate region.

**Figure 9:**
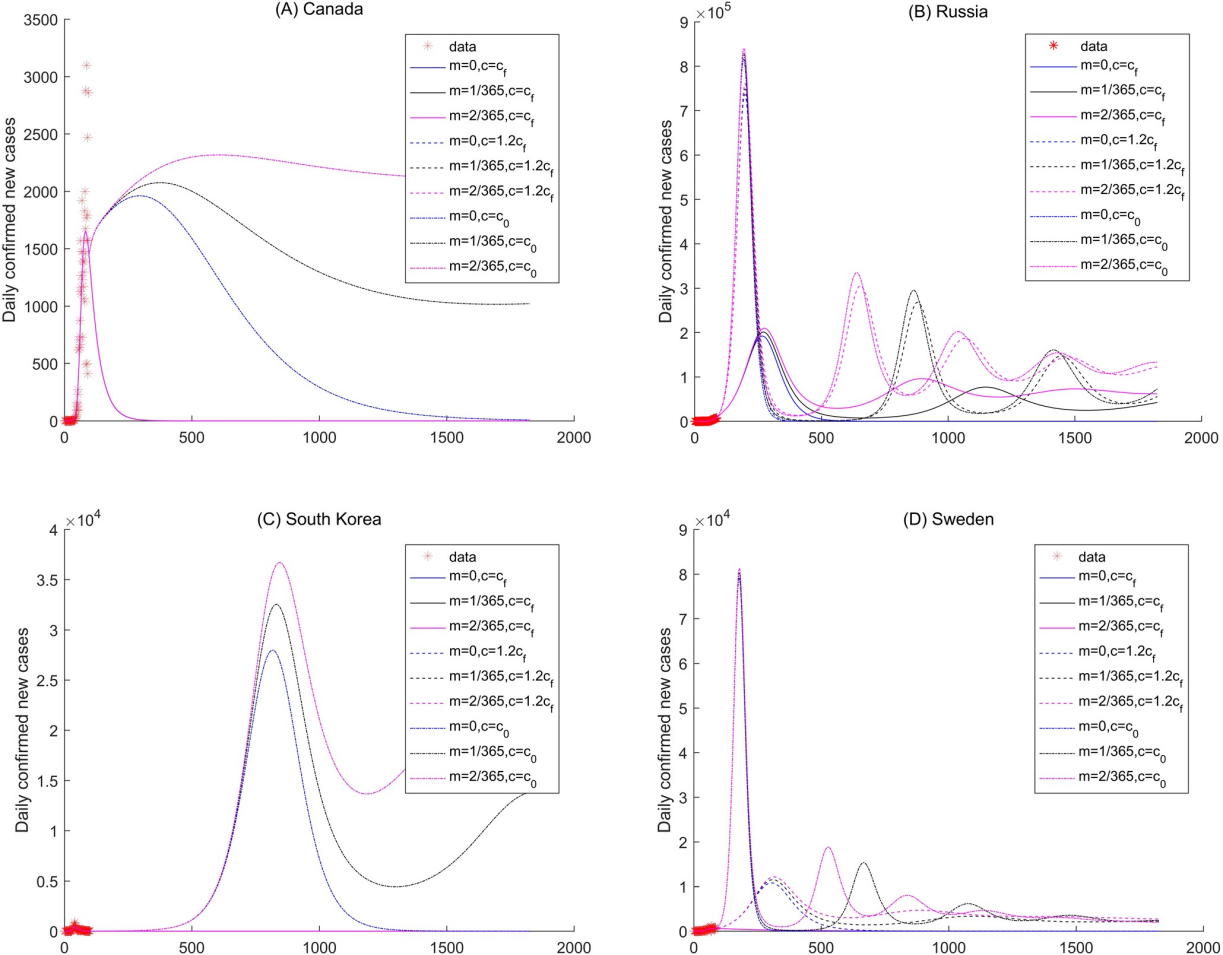
Sensitivity analysis of the daily new confirmed cases of Canada, Russia, South Korea, and Sweden in cold region.

In this section, the future changes of the COVID‐19 pandemic are explored under nine different scenarios with three contact rates, that is, $c=c_{f},\;1.2c_{f}$, and $c_{0}$, indicating the increased contact value, and three immune loss rates, that is, $m=0,\;\frac{1}{365}$, and $\frac{2}{365}$, indicating permanent immunity, one year immunity, and half‐year immunity, respectively. Second outbreak and periodic variations of the COVID‐19 pandemic are detected over the five climate regions. The results are displayed in Figures 6, 7, 8, 9 and S9–S12.

In tropical climate regions, some obvious periodic variations are obtained in Brazil, Colombia, India, Peru, and Singapore under the conditions of most of Scenarios 4–9 (Figures 6b–6g). The number of daily new confirmed cases in Bolivia and The Philippines reach their peak values, and then decrease to zero under the nine scenarios, which indicates that the COVID‐19 disease will be controlled in the two countries in the future (Figures 6a and 6f). Cameroon, Dominican Republic, Ecuador, Nigeria, Panama, and Puerto Rico exhibit periodic variations of the number of new daily confirmed cases with increased contact rates (Figures S9a, S9c, S9d, and S9g–S9i). Cuba, Ghana, Malaysia, and Thailand will control the disease according to the small number of daily new confirmed cases (Figures S8b, S8e, S9f, and S9j). Moreover, the number of daily new confirmed cases in Bolivia, The Philippines, Cuba, Malaysia, and Thailand will become zero in approximately 200 days (i.e., by the end of 2020). However, more than 1,000 days will be needed to control COVID‐19 in Ghana under large contact rates of $c=1,\;2c_{f}$, and $c_{0}$ (Figure S9e).

For the countries in arid climate regions, Chile, Egypt, Mexico, Pakistan, South Africa, Spain, Turkey, and America exhibit multiple periodic variations (Figure 7). Among the aforementioned countries, the periods of Egypt, Mexico, Pakistan, South Africa, Spain, Turkey, and America are larger than 1 year. Except for the peak values of the different scenarios for Spain at the same time points (Figure 7i), the other countries have peak values under the different scenarios with different time points. The proposed model successfully predicted the variations of the number of daily new confirmed cases in China (Figure 7b). The number of daily new cases in Iran and Saudi Arabia will become nearly zero in approximately 200 days (Figures 7d and 7g). The number of daily new cases in Afghanistan, Algeria, Bahrain, Iraq, Israel, Kazakhstan, and Kuwait have regular circulations with multiple periods, which indicates that COVID‐19 will exist in a long‐term period due to the large contact rates mainly caused by the economic recovery (Figure S10).

Except for the number of daily new confirmed cases in Ireland reaching nearly zero in approximately 240 days, the other countries in temperate climate regions have periodic circulations of COVID‐19 pandemic transmissions (Figure 8 and S11). Bangladesh, France, Germany, Italy, Japan, New Zealand, Austria, Belgium, Austria, Belgium, Greece, Guinea, Indonesia, and The Netherlands show that the COVID‐19 pandemic will reach regular circulation within the period of more than 1 year.

In cold climate regions, several countries exhibit periodic variations of the number of daily new confirmed cases, such as Russia, Sweden, and Armenia (Figures 9b and 9d, and S12a). The number of daily new confirmed cases reaches the peak value in a short time period, and then becomes nearly zero under the different scenarios in countries such as Bulgaria and Solvakia (Figures S12d and S12q).

## 5 Discussion

The ongoing COVID‐19 pandemic has rapidly spread in more than 200 countries and has caused 157,289,118 cases leading to 3,277,272 deaths according to the data last updated: 2021/5/9, 4:43 p.m. CEST OF WHO COVID‐19 Dashboard, and poses a severe threat to public health worldwide. The projection of the transmission dynamics of COVID‐19 into the future plays a significant role in devising and implementing prevention and control strategies. In this study, a SEICR model is proposed to investigate the future variations of the COVID‐19 pandemic from nine scenarios based on different immune loss rates and contact rates over five different worldwide climate regions.

In the development and constructer of the general SEICR model, the contact rate and the detection rate are considered. In fact, the detection capacity is mainly determined by the level of the public health system which is largely impacted by the GDP per capita. The contact rate directly reflects the population density. It is a huge challenge for a general model to capture the COVID‐19 variations for all the 84 countries. Moreover, it is well known that more parameters will caused more uncertainties for a model. Three statistic metrics: CC, RB, and DISO are employed to quantify the model performance which suggest that our model can capture the COVID‐19 variations of the 84 countries. Two issues should be clarified and had a further discussion.

### 5.1 Differences of the COVID‐19 Variations Over the Four Climate Regions

The results obtained from our model are objectively obtained according to the COVID‐19 data from the 84 countries. The relationships between the climate factors and the COVID‐19 variations or the roles of the climate changes on the COVID‐19 are not discussed in this study. We only explore whether there exist COVID‐19 transmission differences between the different climate regions. The impacts of the climate factors on the COVID‐19 disease will be investigated in our future work with more datasets and new approaches.

Our results show that temperate and cold climate regions have a larger transmission rate than arid and tropical climate regions, which illustrates that cold and dry conditions may increase the transmission rate of the SARS‐CoV‐2 virus. To further investigate the differences of the COVID‐19 variations over the four climate regions, the differences of the transmission rates and the COVID‐19 variations are tested by the Student’s test. There is no significant difference of the transmission rates of the countries in different climate regions. This fact emphasizes the reality that a COVID‐19 outbreak can occur in temperate and cold climate regions. More factors in a complex system contribute to the rapid transmission, such as contact rate, medical level, and the quality of the public health system (Baker et al., 2020; Hufnagel et al., 2004; Paraskevis et al., 2021). It should also be considered that our results support the limited role of climate on the transmission of COVID‐19 (Baker et al., 2020), rather than cold and dry climates increasing the transmission of the virus, due to the limited data on the current epidemic.

Some recent works try to explore the relationships between climate factors and COVID‐19 pandemic which mainly focus on temperature and humidity (J. Liu et al., 2020; Ma et al., 2020; Meo et al., 2020; Peter et al., 2020; Prata et al., 2020). For example, low temperature, mild diurnal temperature range and low humidity likely favor the transmission of COVID‐19 (J. Liu et al., 2020). A positive association is found between daily death counts of COVID‐19 and diurnal temperature range (DTR). Absolute humidity is negatively associated with daily death counts of COVID‐19 (Ma et al., 2020). A significant decrease in incidence of daily cases and deaths in countries with high temperatures and low humidity (warmest countries), compared to those countries with low temperatures and high humidity (coldest countries) (Meo et al., 2020). But these results have large uncertainties because the COVID‐19 data and climate factor data are insufficient and all the studies only focus on the regional COVID‐19 pandemic (J. Liu et al., 2020; Prata et al., 2020). WHO also pointed that there is currently no conclusive evidence that either weather (short term variations in meteorological conditions) or climate (long‐term averages) have a strong influence on transmission (https://www.who.int/emergencies/diseases/novel-coronavirus-2019/). Therefore, it must employ more data set to investigate the effects of climate factors on the COVID‐19 transmission. Climatic factors affecting COVID‐19 transmission should be cautiously reexamined when the data are sufficient.

### 5.2 Predicted Variations of the Global COVID‐19 Pandemic

In this study, predicted variations of the global COVID‐19 pandemic were discussed based on different contact rates and immune loss rates. The following was assumed: permanent immunity with m=0 and duration of immunities with $m=\frac{1}{365}$ and $m=\frac{2}{365}$ (i.e., one year immunity and half‐year immunity, respectively) in model (2.1) to explore the future COVID‐19 variations. Our results suggest that contact rate plays a key role in controlling the disease, while immunity plays a temporary role. In particular, under the same contact rates, the longer immunity period will be beneficial to disease control, but it cannot control disease extinction. When the immunity to SARS‐CoV‐2 is not permanent, the COVID‐19 pandemic exhibits periodic variabilities in some countries of the five climate regions (e.g., Brazil and India in Figures 5b and 5d, respectively), which indicates that the disease will enter into regular circulation as the most recent conclusion (Kissler et al., 2020). If the immunity to SARS‐CoV‐2 is permanent, the disease could disappear after causing a major outbreak for more than 60 days, such as in Saudi Arabia in Figure 6g.

With the strict disease control measures employed, a small contact rate plays an important role in controlling the COVID‐19 pandemic. However, large contact rates (i.e., $c=1.2c_{f}$ and $c=c_{0}$) will result in COVID‐19 fluctuations, including some obvious multiple periods, such as in Egypt (Figure 6c), Mexico (Figure 6e), and Germany (Figure 7c). This result suggests that decreasing the contact rate based on the non‐pharmaceutical interventions is the most effective means to reduce worldwide transmission of SARS‐CoV‐2, for example, by maintaining safe physical distancing, closing schools and workplaces, limiting the sizes of gatherings, wearing face coverings and eye protection, and instituting community quarantines (Chu et al., 2020; Hu et al., 2020; Li et al., 2020).

**Figure 10:**
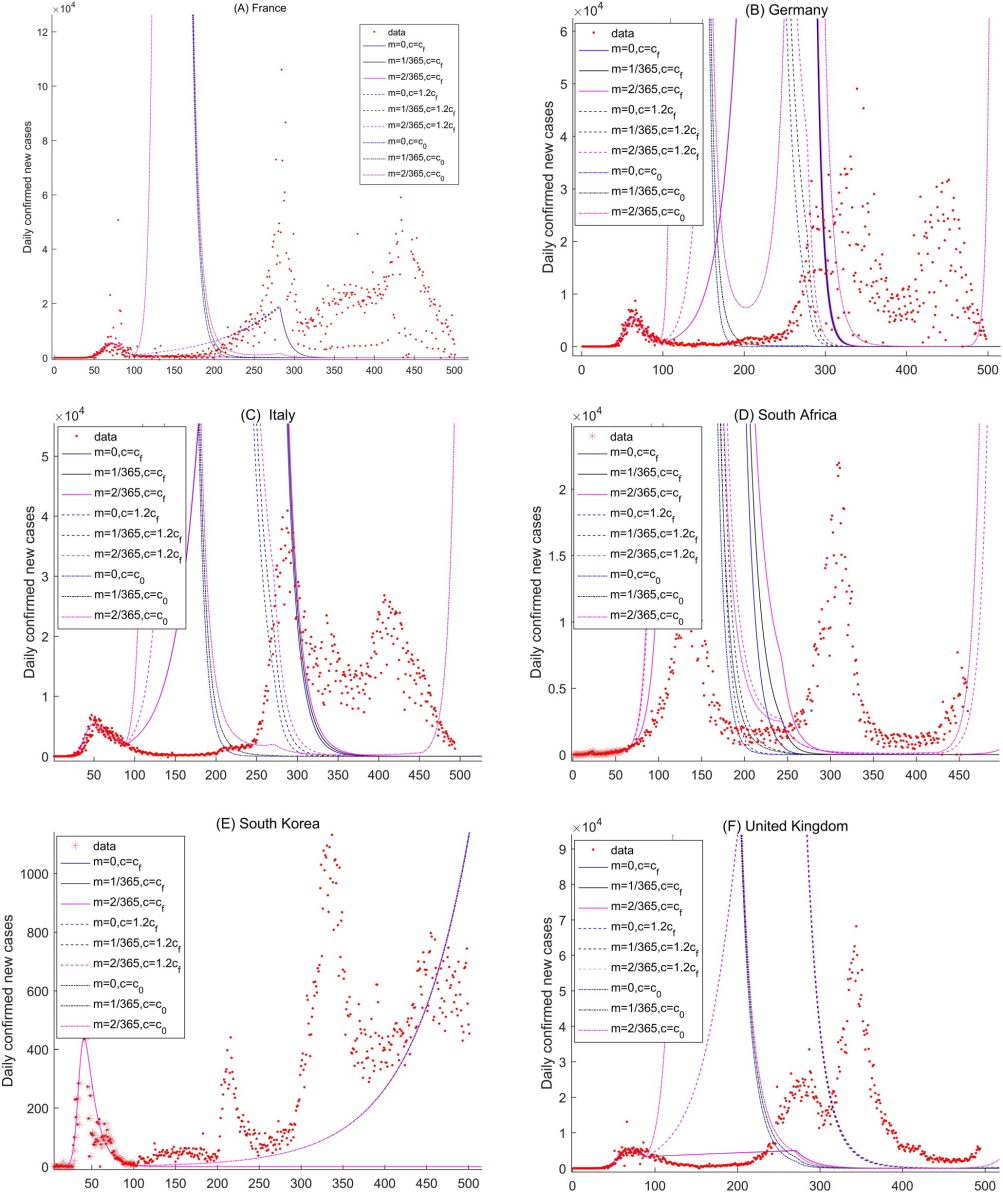
Comparisons of the long term daily new confirmed cases between the actual more than one year variations and the potential variations obtained by model (2.1) for six countries: France, Germany, Italy, South Africa, South Korea, and United Kingdom. The comparison period is from the first data to the June 7, 2021.

In this study, although we only investigate the potential variations at nine different scenarios, a comparison of the daily confirmed new cases between the actual more than one year variations and the potential variations obtained by our model. The comparison is analyzed at six countries: France, Germany, Italy, South Africa, South Korea, and United Kingdom in Figure 10. The comparison period is from the first data to the June 7, 2021. The predicted variations of France are well agreed with the actual variations for about 200 days (Figure 10a) at S1 scenario. For Germany, Italy, South Korea, and United Kingdom, the well captured variations are for about 100 days (Figures 10b, 10C, 10e, and 10f). For a longer period, the prediction of our model cannot well capture the actual variations because of the only about 60 days time series used in the parameter estimations. However, the long term variations are general captured by our model in France, Italy and South Africa from Figures 10a, 10c, and 10d. Indeed, the long term simulation or prediction about the COVID‐19 pandemic should be discussed in a new model because the COVID‐19 variations are changed timely resulted by many complex factors.

Since the first COVID‐19 case was reported, all the countries and regions of the world have been affected, and peoples’ way of life has changed. Comprehensive strategies have been developed to fight against the COVID‐19 pandemic by each country based on their specific epidemiological situations, capacities, and the capabilities of their public health systems, especially for low‐ and middle‐income countries. Our findings suggest that this pandemic will spread over all five climate regions in the future which are proved by the present COVID‐19 pandemic variations in the world.

The effective strategy to date has been to decrease contact with COVID‐19 sufferers, and the reduction of contact rate can help prevent the COVID‐19 pandemic from taxing the capacity of public health systems across the globe. Non‐pharmaceutical interventions are always the effective strategy in control and prevention the COVID‐19 which will may eliminate the COVID‐19 pandemic completely together with the roles of the vaccines.

## Conflict of Interest

The authors declare no competing interests.

## Supporting information

Supporting Information S1

Table S1

## Data Availability

The COVID‐19 data are sourced from WHO (https://covid19.who.int/). The Köppen‐Geiger climate classification data are from Beck et al. (2018).
