# Supplementary material for: Dynamical Variations of the Global COVID‐19 Pandemic Based on a SEICR Disease Model: A New Approach of Yi Hua Jie Mu
Source: Geohealth. 2021 Aug 1;5(8):e2021GH000455. doi: 10.1029/2021GH000455 (PMC8381858; doi:10.1029/2021GH000455)
Supplement: Supplementary file 1 — Supporting Information S1 [file GH2-5-e2021GH000455-s001.pdf]

Supplementary materials for
Dynamical variations of the Global COVID-19 Pandemic based on a SEICR disease model: a new approach of Yi Hua Jie Mu

Xia Wang[1], Gang Yin[2], Zengyun Hu[3,4]*, Daihai He[5], Qianqian Cui[6], Xiaomei Feng[7], Zhidong Teng[8], Qi Hu[9], Jiansen Li[10], Qiming Zhou[11]

[1]School of Mathematics and Information Science, Shaanxi Normal University, Xian 710119, China

[2]College of Resource and Environment Science, Xinjiang University, Urumqi, Xinjiang 830046, China

[3]State Key Laboratory of desert and Oasis Ecology, Xinjiang Institute of Ecology and Geography, Chinese Academy of Sciences, Urumqi, Xinjiang 830011, China

[4]Research Center for Ecology and Environment of Central Asia, Chinese Academy of Sciences, Urumqi, Xinjiang 830011, China

[5]Department of Applied Mathematics, Hong Kong Polytechnic University, Hong Kong SAR China

[6]School of Mathematics and Statistics, Ningxia University, Yinchuan, Ningxia 750021, China

[7]School of Mathematics and Informational Technology, Yuncheng University, Yuncheng 044000, China

[8]College of Mathematics and System Sciences, Xinjiang University, Urumqi, Xinjiang 830046, China

[9]School of Natural Resources and Department of Earth and Atmospheric Sciences, University of Nebraska Lincoln, Lincoln, Nebraska

[10]Guangdong Provincial Center for Disease Control and Prevention, Guangzhou 511430, China

[11] Department of Geography, Hong Kong Baptist University, Hong Kong, China

---

*Corresponding author. Zengyun Hu, email: huzengyun@ms.xjb.ac.cn;

Text S Background of the COVID-19 pandemic

For the spatial diffusions of the COVID-19 pandemic, different countries have significant differences that mainly result from economic restart and increased testing capability (Figure S1). On April 3, 2020, the confirmed cases were mainly distributed over the NH, while the tropical regions of the NH and the SH had a small proportion of confirmed cases. In particular, the United States of America (America), Spain, and Italy had confirmed COVID-19 cases numbering greater than 100,000, i.e., 254,556, 117,710, and 115,242 cases, respectively. The countries with confirmed cases numbering between 80,000 and 100,000 were China (82,875) and Germany (85,122). The countries within Central Asia, Mongolia, and the countries in Africa (except south Africa) had confirmed cases numbering fewer than 1,000 (Figure S1A).

Except for America, Spain, and Italy, the new countries with numbers of confirmed cases greater than 100,000 on April 27, 2020 were Germany (increased from 85,122 to 159,103) and France (increased from 59,929 to 162,220), and the total number of confirmed cases worldwide (2,026,027) was larger than 2 million by the same date (Figure S1B). In fact, America (increased from 254,556 to 987,916), Germany, and France contributed to more than 90% of the increase in confirmed cases. There was no significant increase in South America and Africa at this time.

It only took 12 d for the number of worldwide confirmed cases to increase from 2 million to 4 million by May 9, 2020. The new countries with numbers of confirmed cases larger than 100,000 at this point were Russia (198,676), Turkey (135,569), Iran (106,220), and Brazil (147,003) (Figure S1C). America had the largest number of confirmed cases, i.e., 1,324,352 at this point.

Peru and India were the newest countries with confirmed cases numbering more than 100,000 on May 30, 2020, at which time the total number of confirmed cases worldwide was 6,069,385 (Figure S1D). On June 15 and 28, 2020, the total number of worldwide confirmed cases reached 8,035,398 and 10,138,506, respectively (Figures S1E and S1F, respectively). More than 15 countries had confirmed cases numbering greater than 100,000 at this point, including Brazil, Peru, and South Africa in the Southern Hemisphere (SH) (Figure S1F). America had the largest number of confirmed cases at this point, i.e., 2,597,742, followed by Brazil with 1,319,385 confirmed cases.

Figure Captions

Figure S1: Spatial distributions of the cumulative confirmed COVID-19 cases over the world, for the numbers of 1 million cases (A), 2 millions cases (B), 4 millions cases (C), 6 millions cases (D), 8 millions cases (E), and 10 millions cases (F).

Figure S2: Simulation results of the cumulative confirmed cases, cumulative recovered cases, and cumulative deaths of Bolivia, Brazil, Cameroon, Colombia, Cuba, Dominican Republic, Ecuador, Ghana, India, Malaysia, Nigeria, Panama, Peru, Philippines, Puerto Rico, Singapore and Thailand in tropical region.

Figure S3: Simulation results of the cumulative confirmed cases, cumulative recovered cases, and cumulative deaths of Afghanistan, Algeria, Argentina, Australia, Azerbaijan, Bahrain, Chile, China, Djibouti, Egypt, Iran, Iraq, Israel, Kazakhstan, Kuwait, Mexico, Morocco, Oman, Pakistan, Qatar, Saudi Arabia, South Africa, Spain, Turkey, United Arab Emirates, United States and Uzbekistan in arid region.

Figure S4: Simulation results of the cumulative confirmed cases, cumulative recovered cases, and cumulative deaths of Austria, Bangladesh, Belgium, France, Germany, Greece, Guinea, Indonesia, Ireland, Italy, Japan, Luxembourg, Netherlands, New Zealand, Portugal and United Kingdom in temperate region.

Figure S5: Simulation results of the cumulative confirmed cases, cumulative recovered cases, and cumulative deaths of Armenia, Belarus, Bosnia and Herzegovina, Bulgaria, Canada, Croatia, Czech Republic, Denmark, Estonia, Finland, Hungary, Lithuania, Moldova, Norway, Poland, Romania, Russia, Serbia, Slovakia, Slovenia, South Korea, Sweden, Switzerland and Ukraine in cold region.

Figure S6: Simulation results of the cumulative confirmed cases, cumulative recovered cases, and cumulative deaths of Iceland in polar region.

Figure S7: Distribution of the contact rates c_0 at early transmission period of the 85 countries.

Figure S8: Same as Figure S6, but for the minimum contact rates c_f.

Figure S9: Sensitivity analysis of the daily new confirmed cases of Cameroon, Cuba, Dominican Republic, Ecuador, Ghana, Malaysia, Nigeria, Panama, Puerto Rico, and Thailand in tropical region.

Figure S10: Sensitivity analysis of the daily new confirmed cases of Afghanistan, Algeria, Argentina, Austrialia, Azerbaijan, Bahrain, Djibouti, Iraq, Israel, Kazakstan, Kuwait, Morocco, Oman, Qatar, United Arab Emirates and Uzbekistan in arid region.

Figure S11: Sensitivity analysis of the daily new confirmed cases of Austria, Belgium, Greece, Guinea, Indonesia, Ireland, Luxembourg, Netherlands, and Portugal in temperate region.

Figure S12: Sensitivity analysis of the daily new confirmed cases of Armenia, Belarus, Bosnia and Herzegovina, Bulgaria, Croatia, Czech Republic, Denmark, Estonia, Finland, Hungary, Lithuania, Moldova, Norway, Poland, Romania, Servia, Slovakia, Slovenia, Switzerland, and Ukraine in cold region.

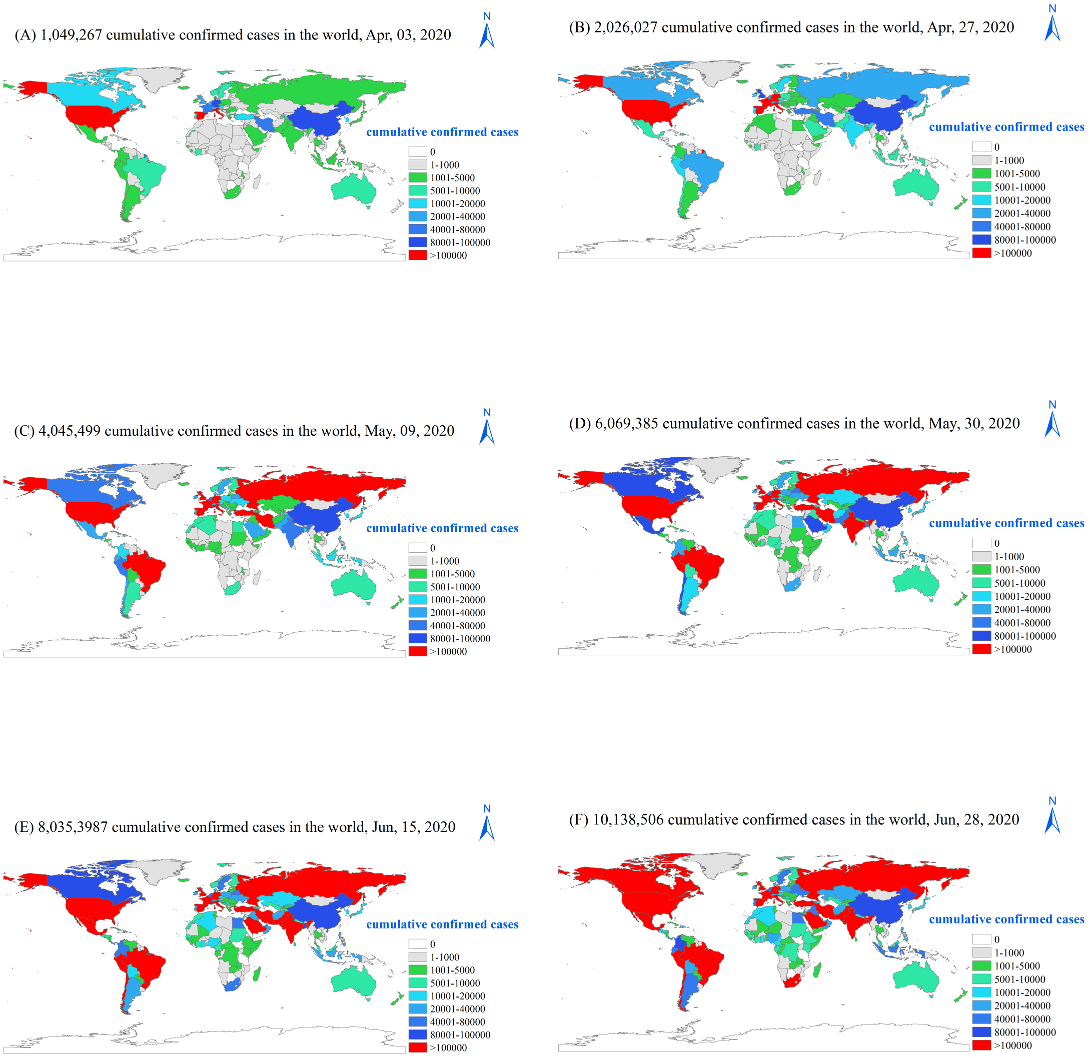

Figure S1: Spatial distributions of the cumulative confirmed COVID-19 cases over the world, for the numbers of 1 million cases (A), 2 millions cases (B), 4 millions cases (C), 6 millions cases (D), 8 millions cases (E), and 10 millions cases (F).

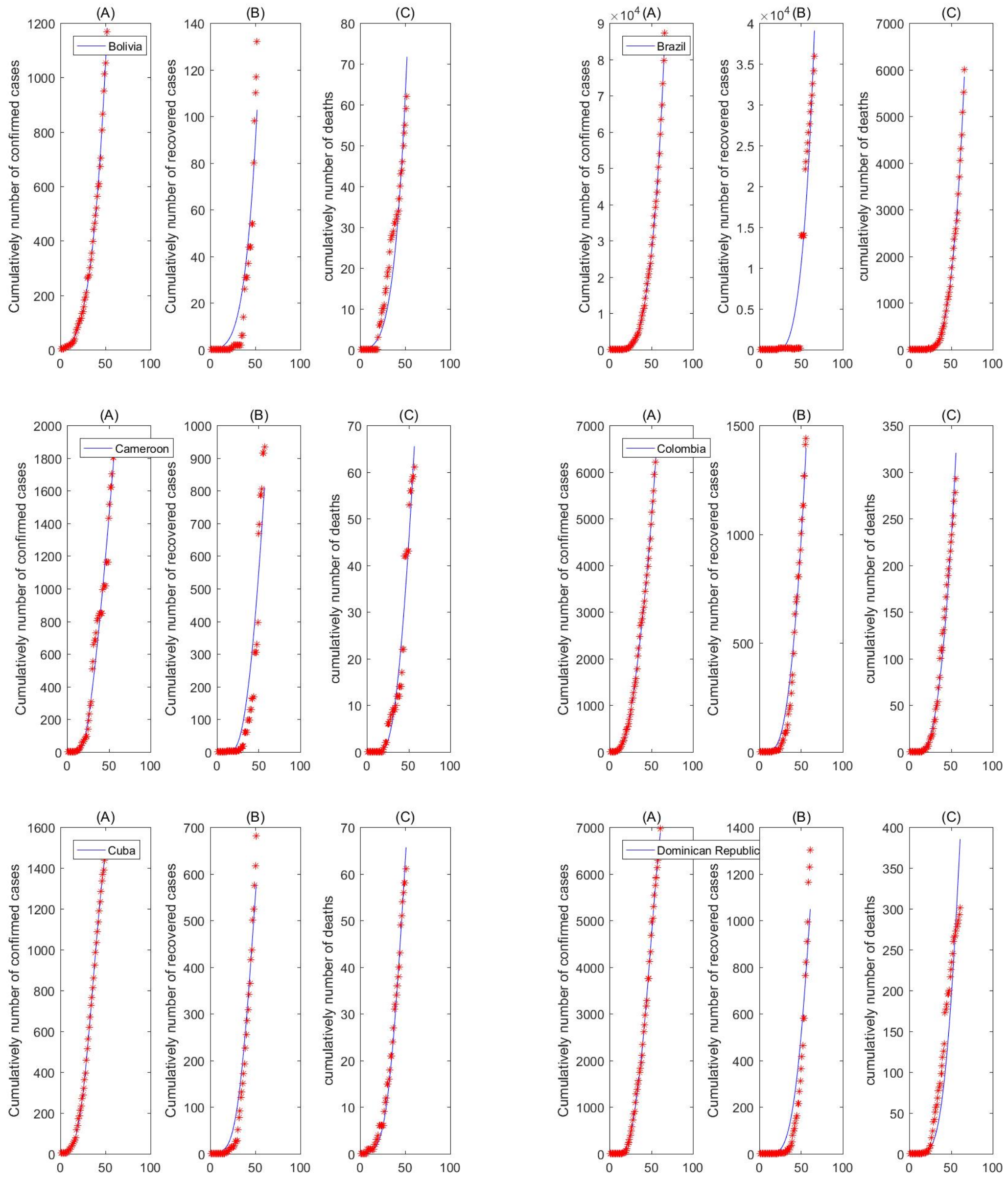
(A)
(B)
(C)
Bolivia
Brazil
Cameroon
Colombia
Cuba
Dominican Republic
Cumulatively number of confirmed cases
Cumulatively number of recovered cases
cumulatively number of deaths
$\times 10^4$

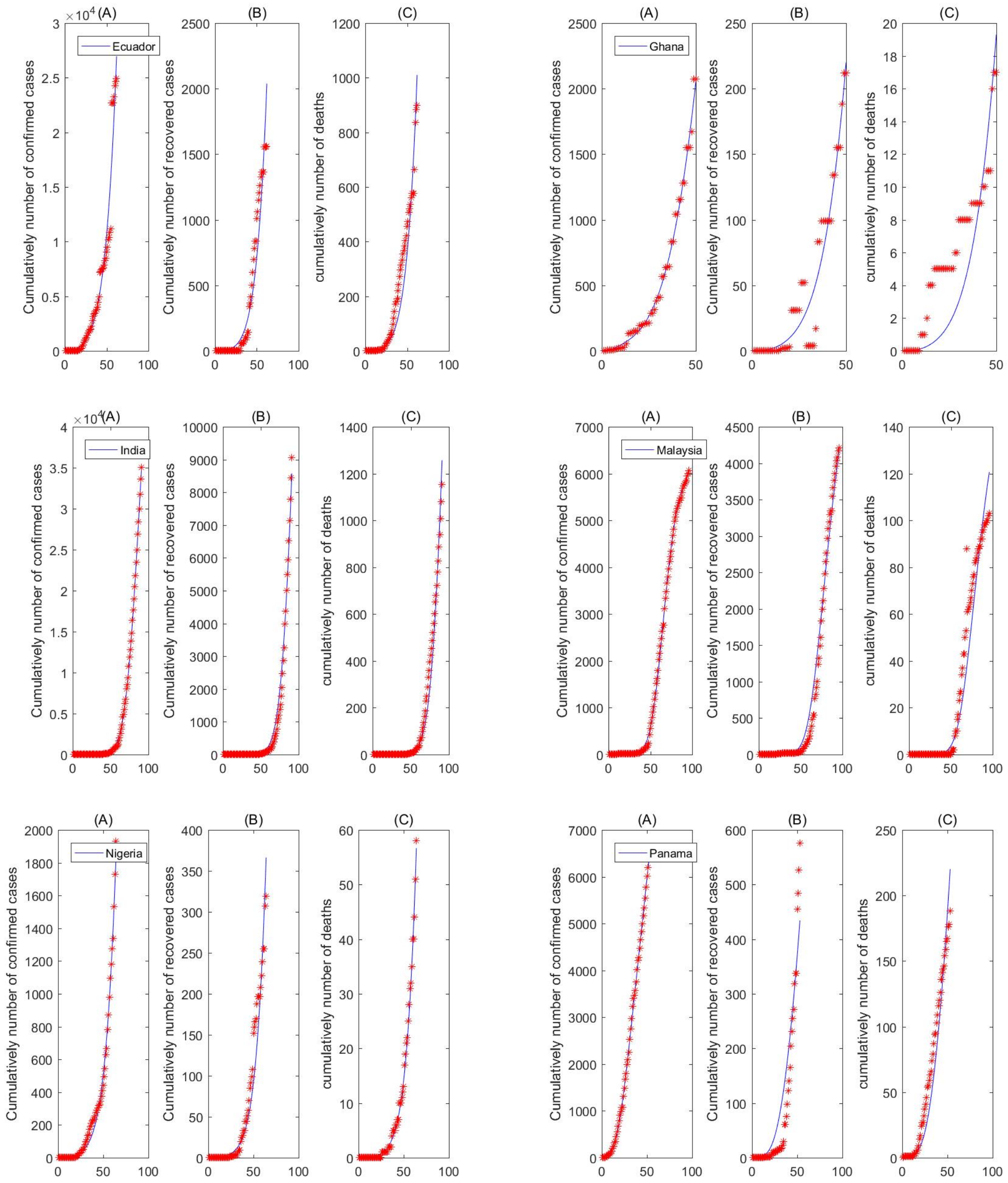
(A)
(B)
(C)
Ecuador
Ghana
India
Malaysia
Nigeria
Panama
Cumulatively number of confirmed cases
Cumulatively number of recovered cases
cumulatively number of deaths
×10^4

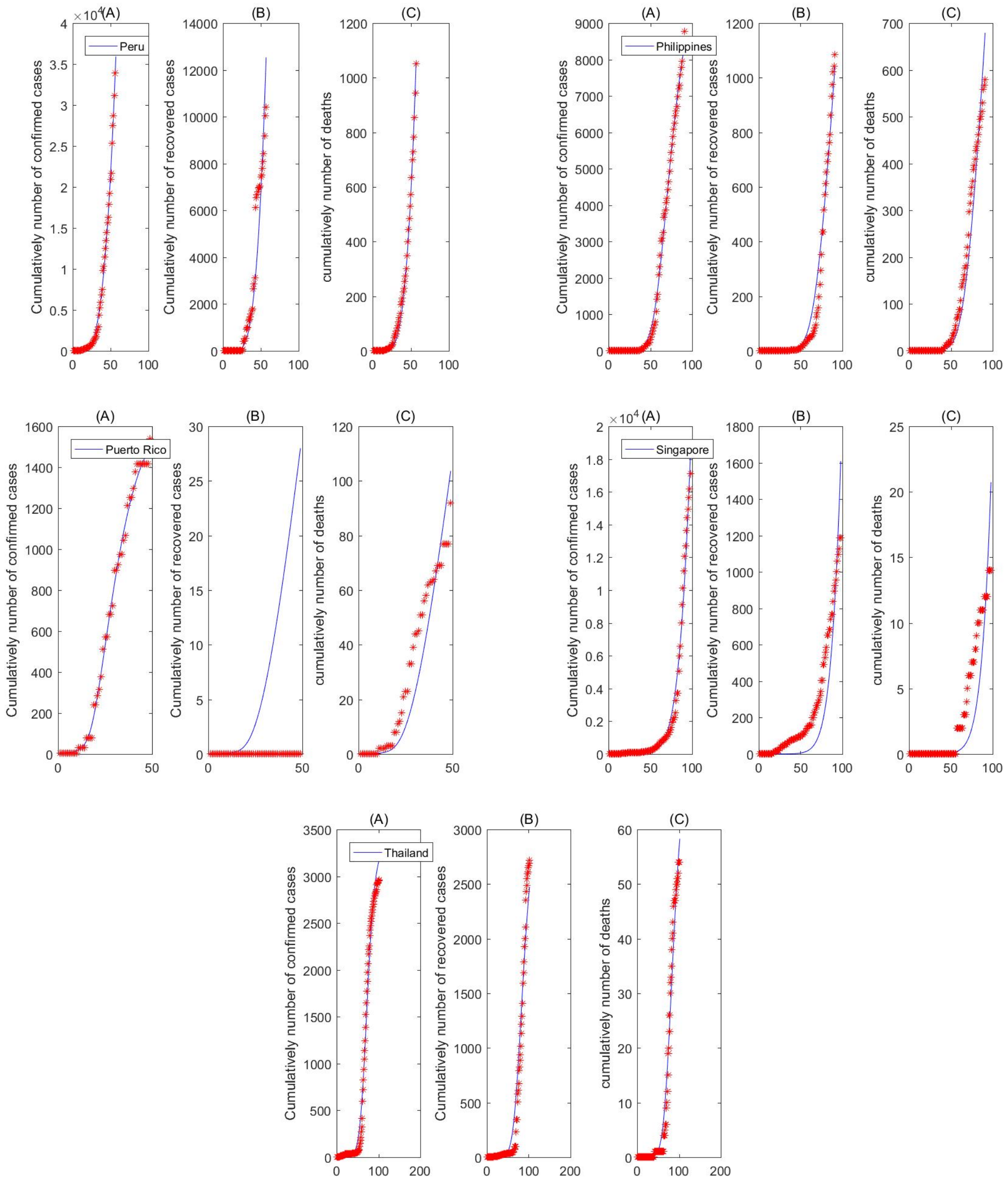

Figure S2: Simulation results of the cumulative confirmed cases, cumulative recovered cases, and cumulative deaths of Bolivia, Brazil, Cameroon, Colombia, Cuba, Dominican Republic, Ecuador, Ghana, India, Malaysia, Nigeria, Panama, Peru, Philippines, Puerto Rico, Singapore and Thailand in tropical region.

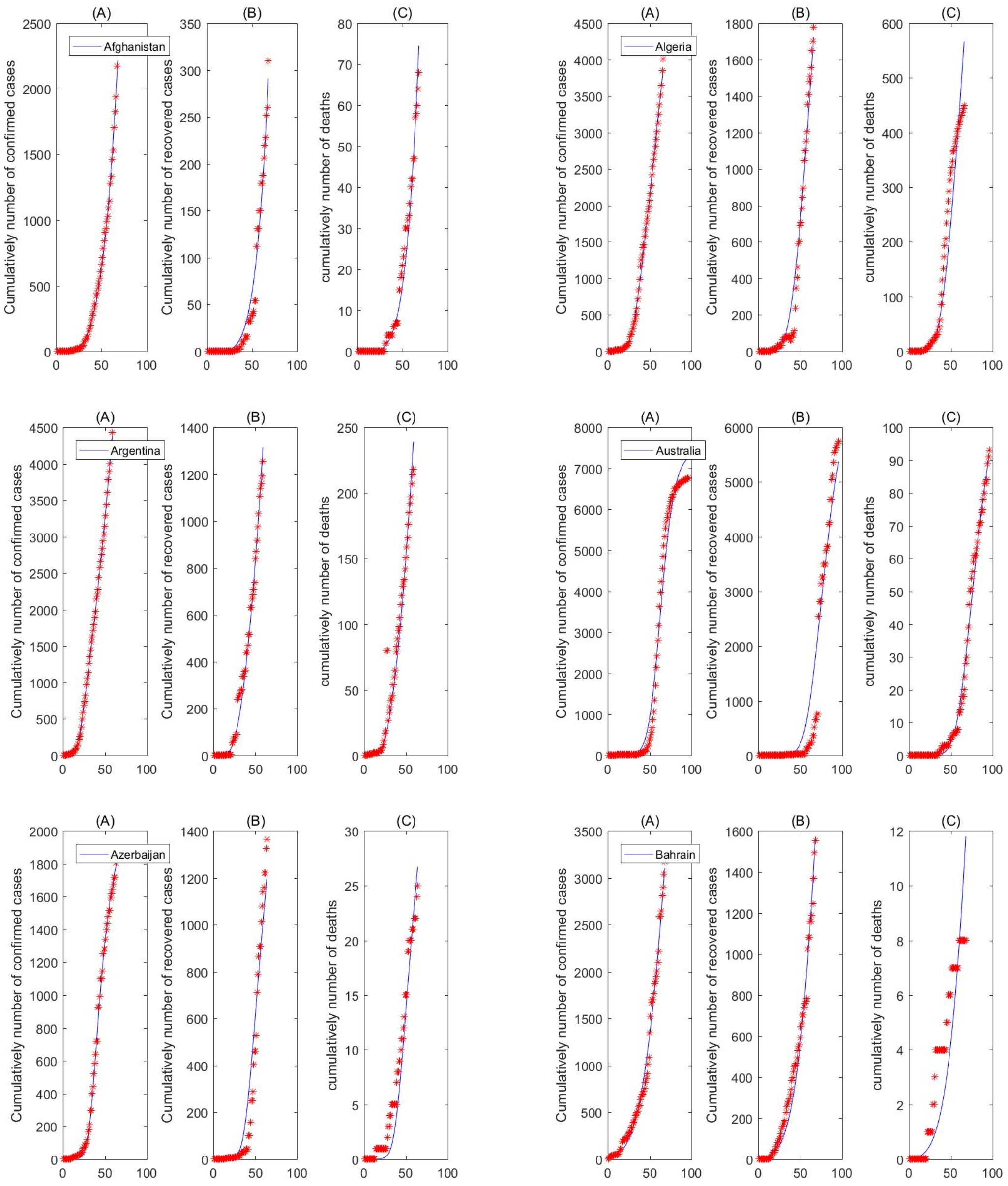
(A)
(B)
(C)
Afghanistan
Algeria
Argentina
Australia
Azerbaijan
Bahrain
Cumulatively number of confirmed cases
Cumulatively number of recovered cases
cumulatively number of deaths

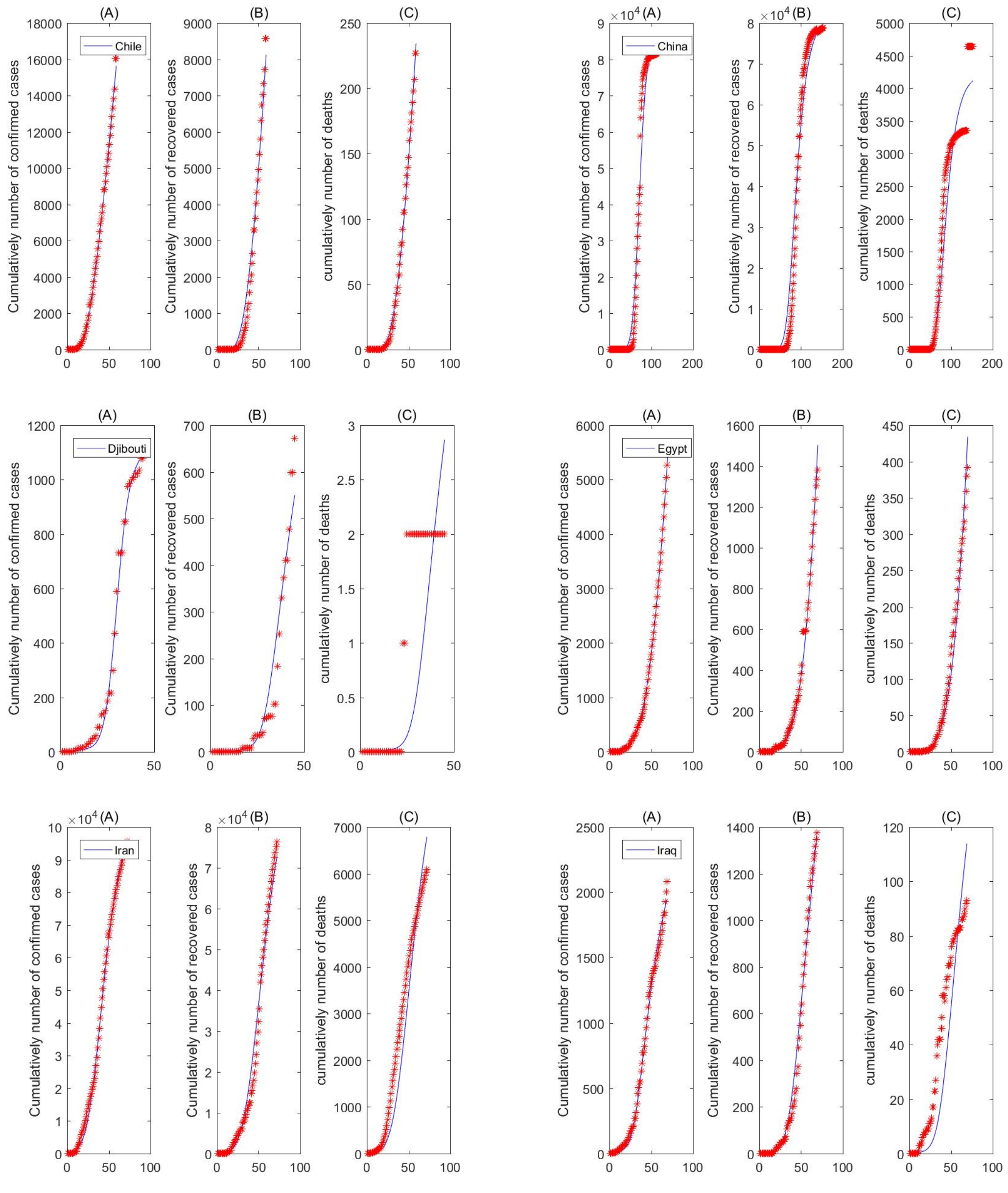
(A)
(B)
(C)
Chile
China
Djibouti
Egypt
Iran
Iraq
Cumulatively number of confirmed cases
Cumulatively number of recovered cases
cumulatively number of deaths
×10^4

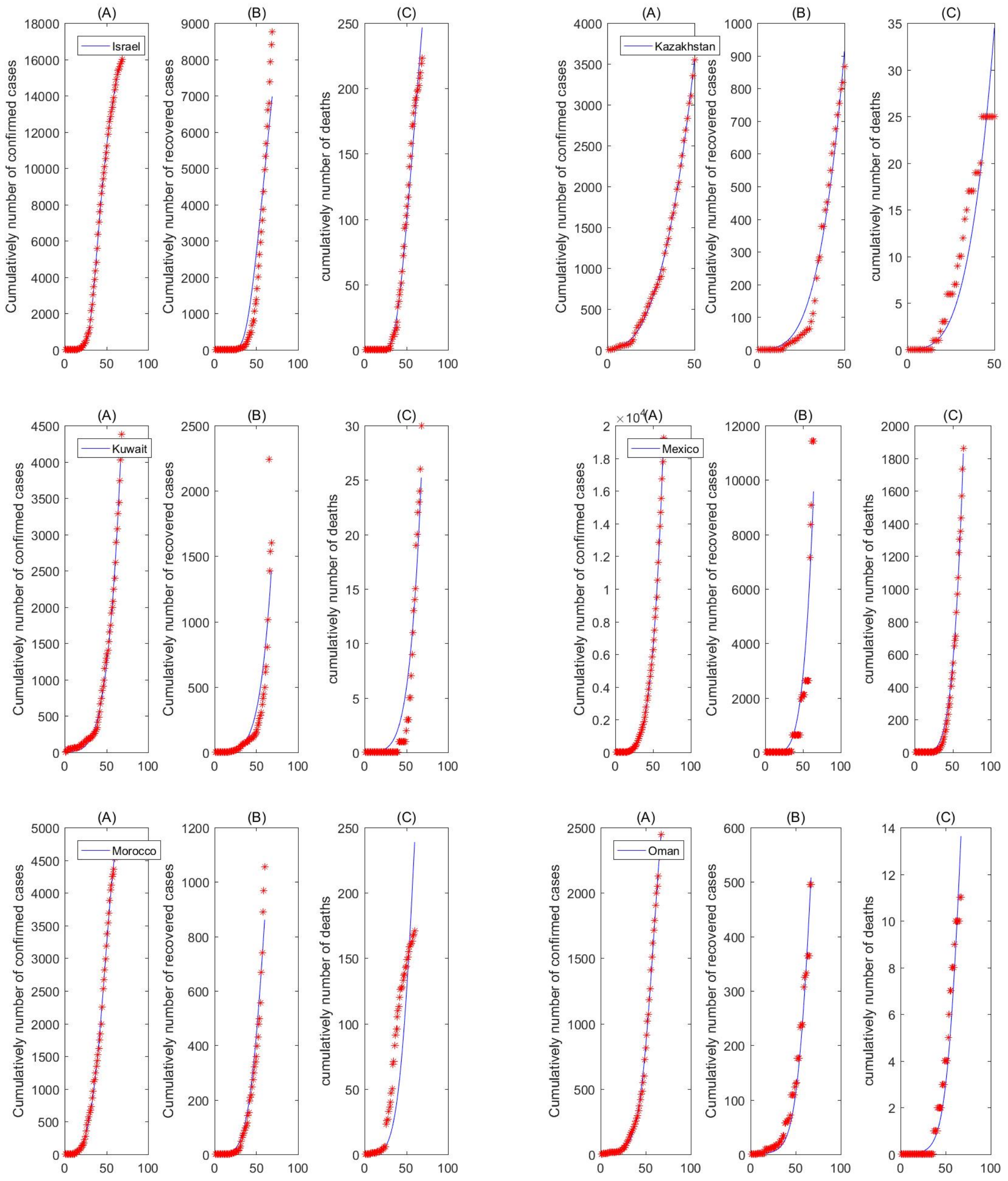
(A)
(B)
(C)
Israel
Kazakhstan
Kuwait
Mexico
Morocco
Oman
Cumulatively number of confirmed cases
Cumulatively number of recovered cases
cumulatively number of deaths

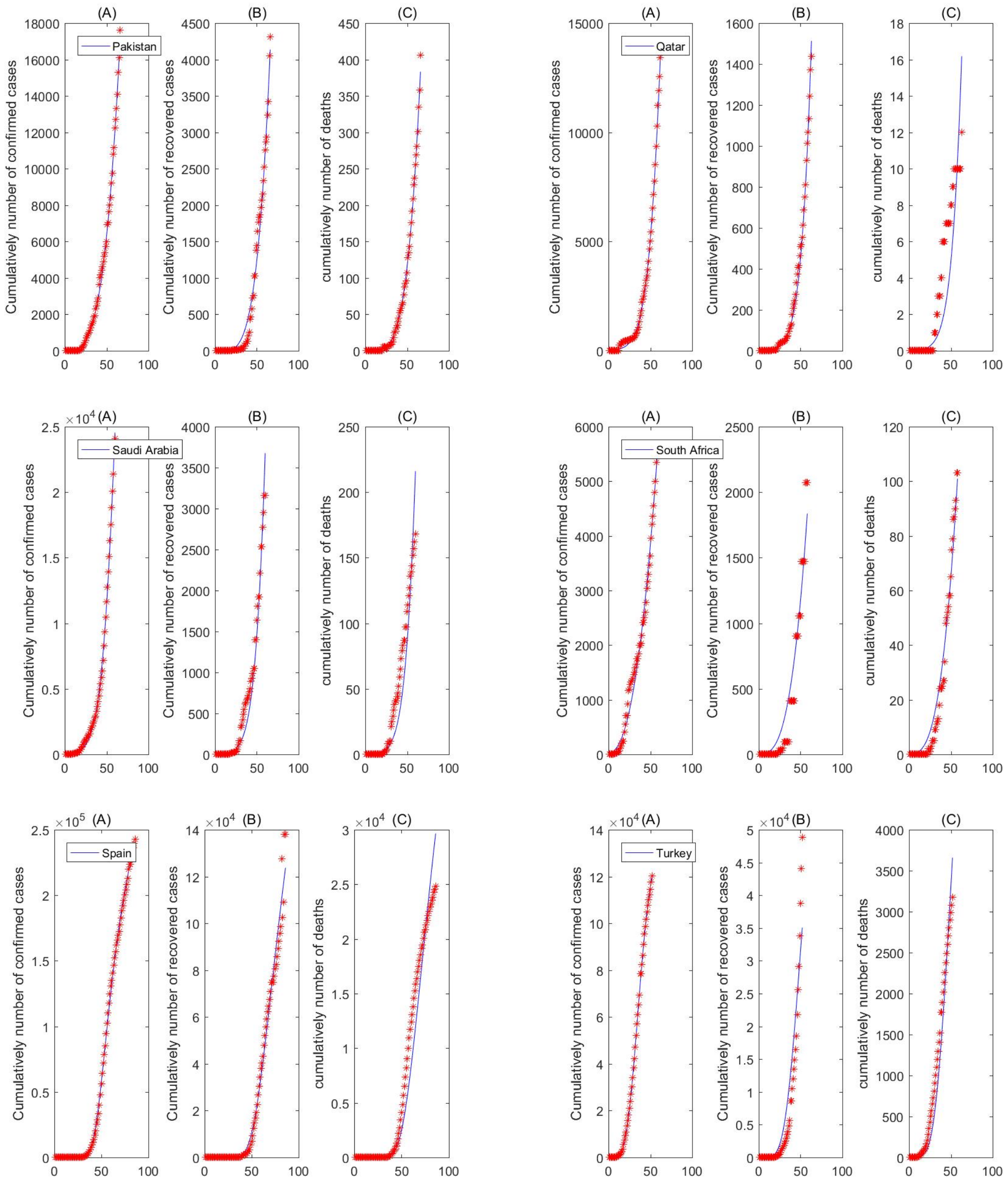
(A)
(B)
(C)
Pakistan
Qatar
Saudi Arabia
South Africa
Spain
Turkey
Cumulatively number of confirmed cases
Cumulatively number of recovered cases
cumulatively number of deaths

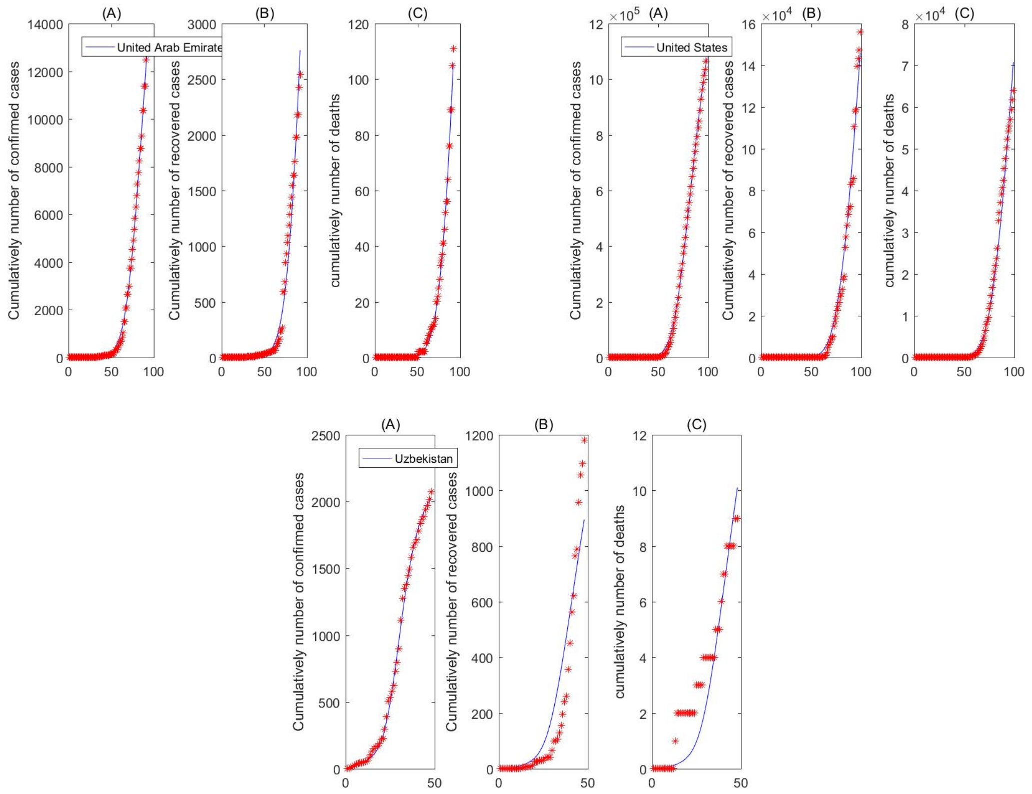

Figure S3: Simulation results of the cumulative confirmed cases, cumulative recovered cases, and cumulative deaths of Afghanistan, Algeria, Argentina, Australia, Azerbaijan, Bahrain, Chile, China, Djibouti, Egypt, Iran, Iraq, Israel, Kazakhstan, Kuwait, Mexico, Morocco, Oman, Pakistan, Qatar, Saudi Arabia, South Africa, Spain, Turkey, United Arab Emirates, United States and Uzbekistan in arid region.

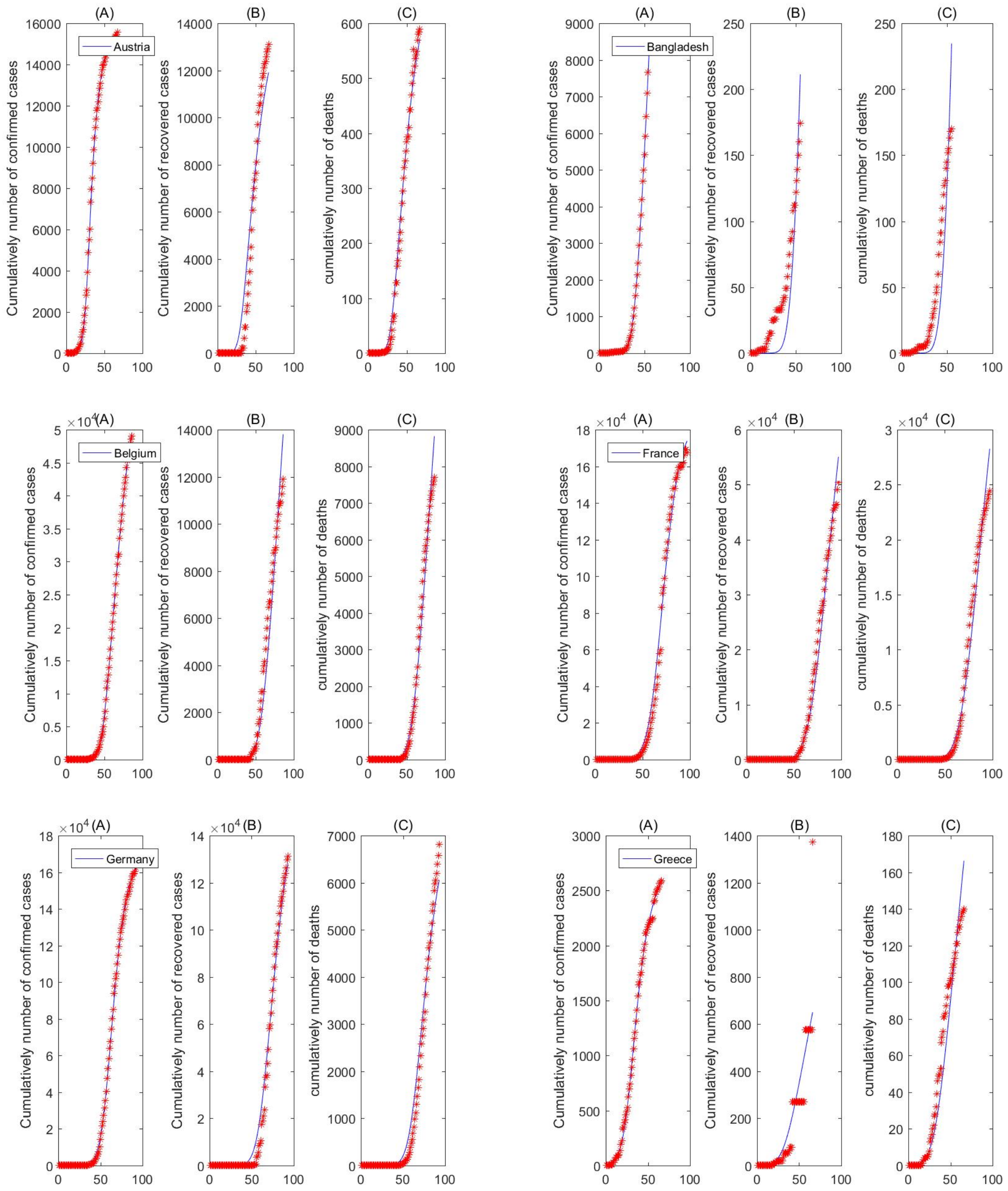
(A)
(B)
(C)
Austria
Bangladesh
Belgium
France
Germany
Greece
Cumulatively number of confirmed cases
Cumulatively number of recovered cases
cumulatively number of deaths
×10^4

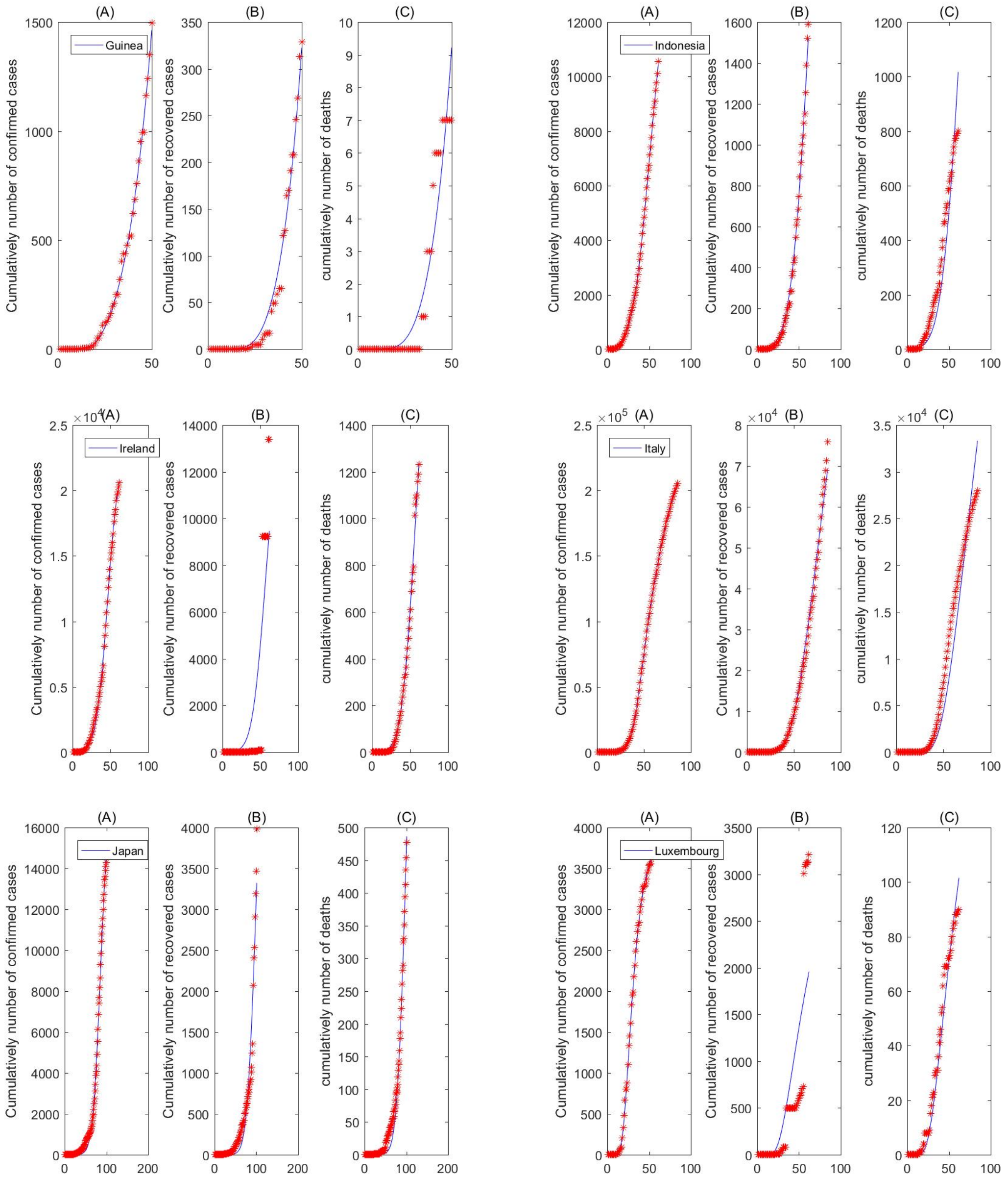
(A)
(B)
(C)
Guinea
Indonesia
Ireland
Italy
Japan
Luxembourg
Cumulatively number of confirmed cases
Cumulatively number of recovered cases
cumulatively number of deaths
×10^4
×10^5
×10^4
×10^4

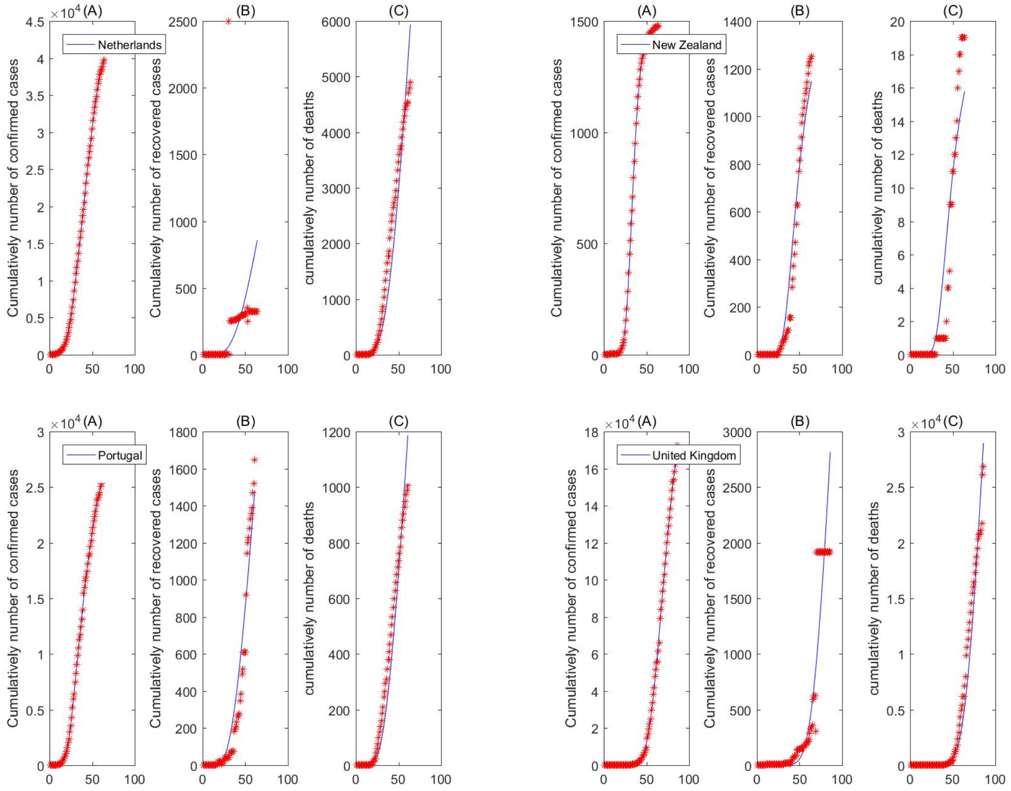

Figure S4: Simulation results of the cumulative confirmed cases, cumulative recovered cases, and cumulative deaths of Austria, Bangladesh, Belgium, France, Germany, Greece, Guinea, Indonesia, Ireland, Italy, Japan, Luxembourg, Netherlands, New Zealand, Portugal and United Kingdom in temperate region.

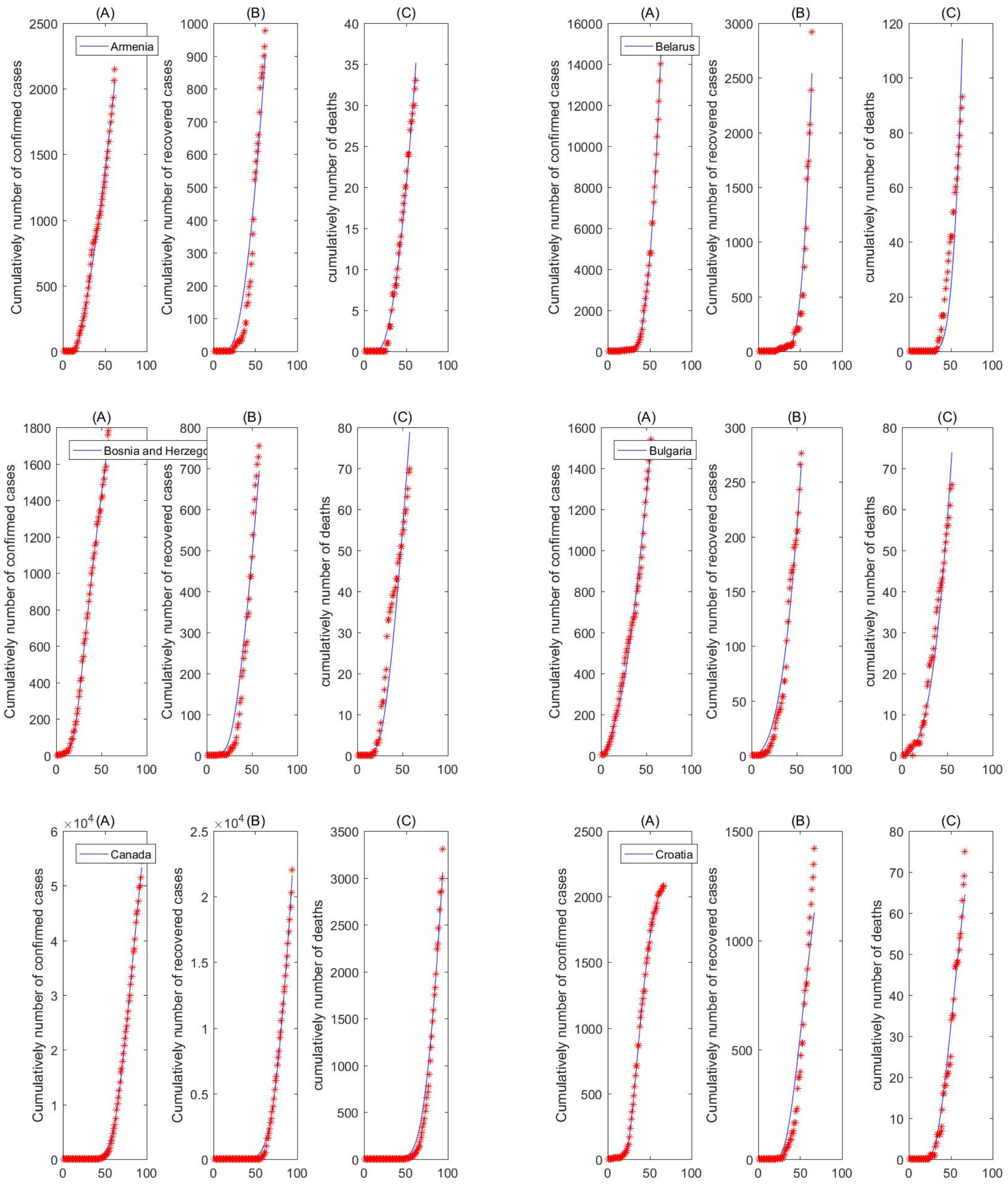
(A)
(B)
(C)
Armenia
Belarus
Bosnia and Herzego
Bulgaria
Canada
Croatia
Cumulatively number of confirmed cases
Cumulatively number of recovered cases
cumulatively number of deaths
×10^4

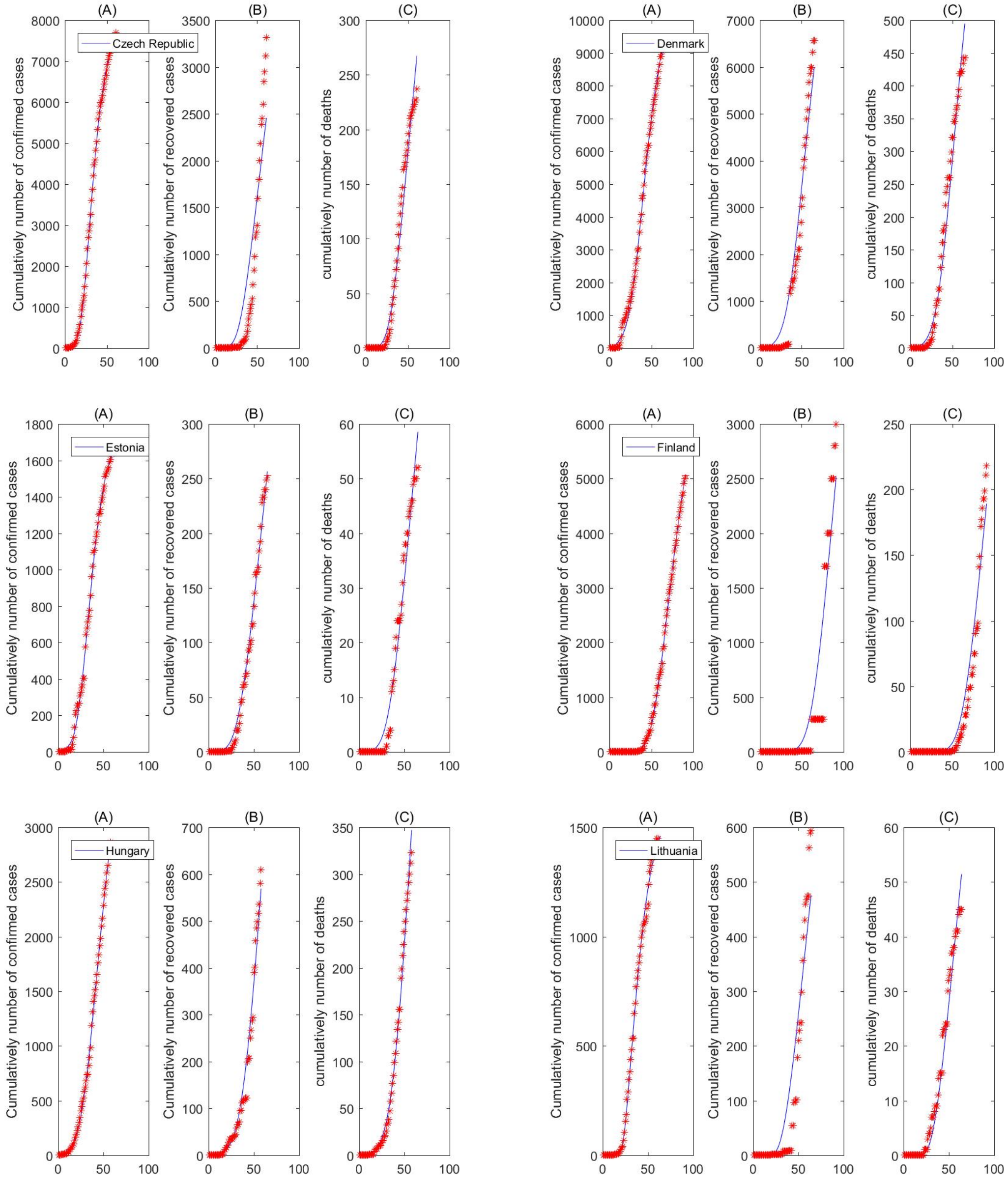
(A)
(B)
(C)
Czech Republic
Denmark
Estonia
Finland
Hungary
Lithuania
Cumulatively number of confirmed cases
Cumulatively number of recovered cases
cumulatively number of deaths

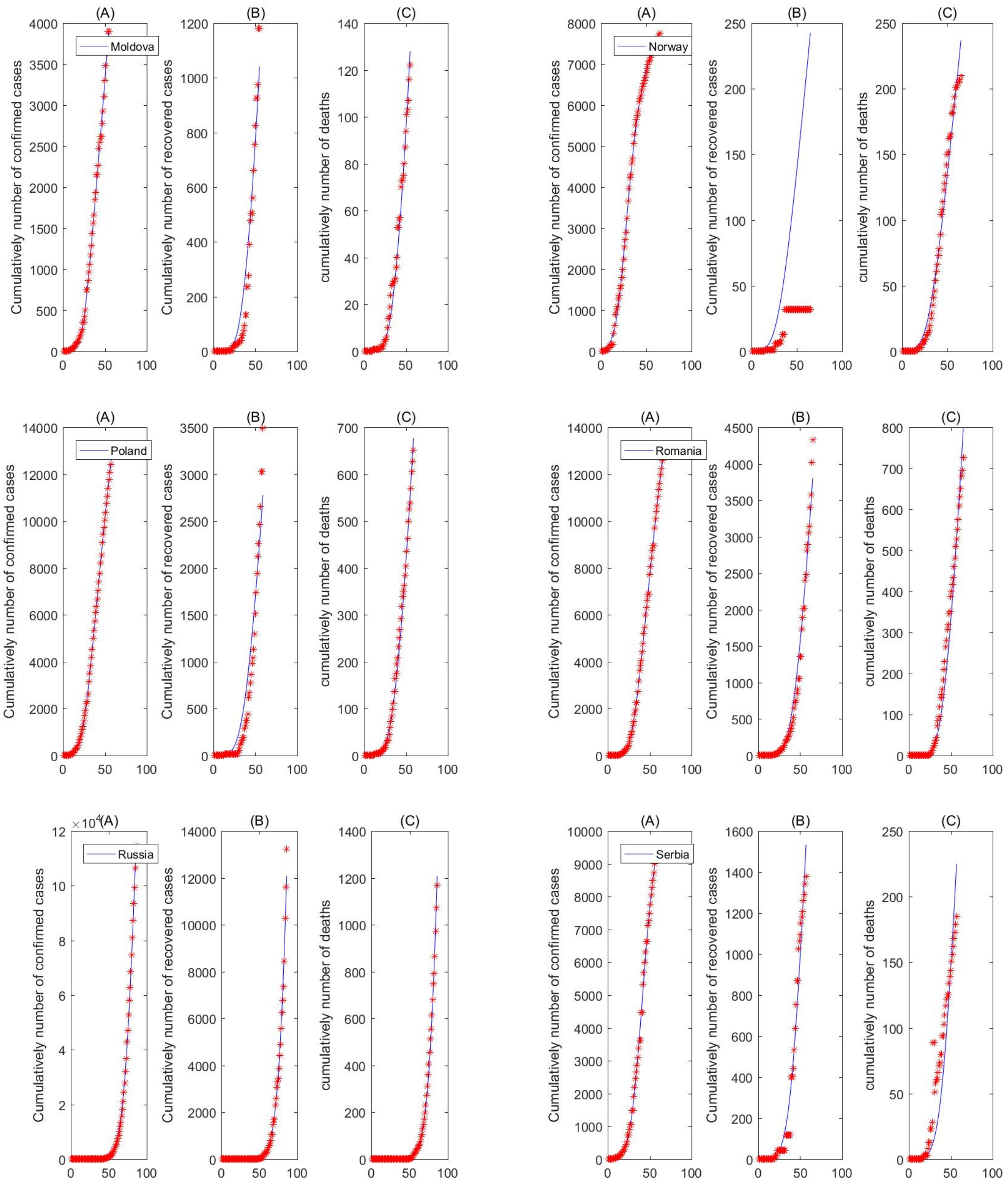
(A)
(B)
(C)
Moldova
Norway
Poland
Romania
Russia
Serbia
Cumulatively number of confirmed cases
Cumulatively number of recovered cases
cumulatively number of deaths
×10^4

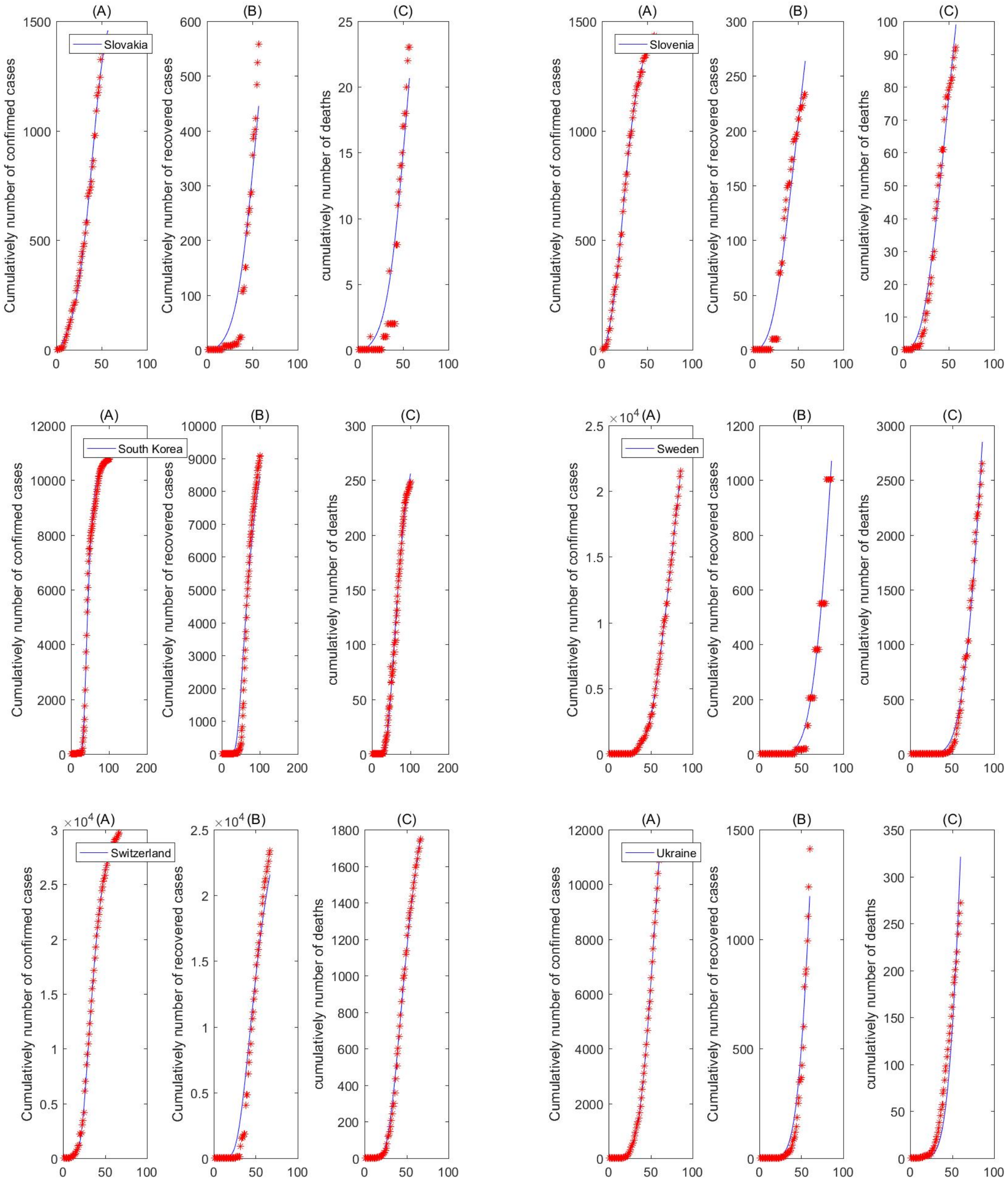

Figure S5: Simulation results of the cumulative confirmed cases, cumulative recovered cases, and cumulative deaths of Armenia, Belarus, Bosnia and Herzegovina, Bulgaria, Canada, Croatia, Czech Republic, Denmark, Estonia, Finland, Hungary, Lithuania, Moldova, Norway, Poland, Romania, Russia, Serbia, Slovakia, Slovenia, South Korea, Sweden, Switzerland and Ukraine in cold region.

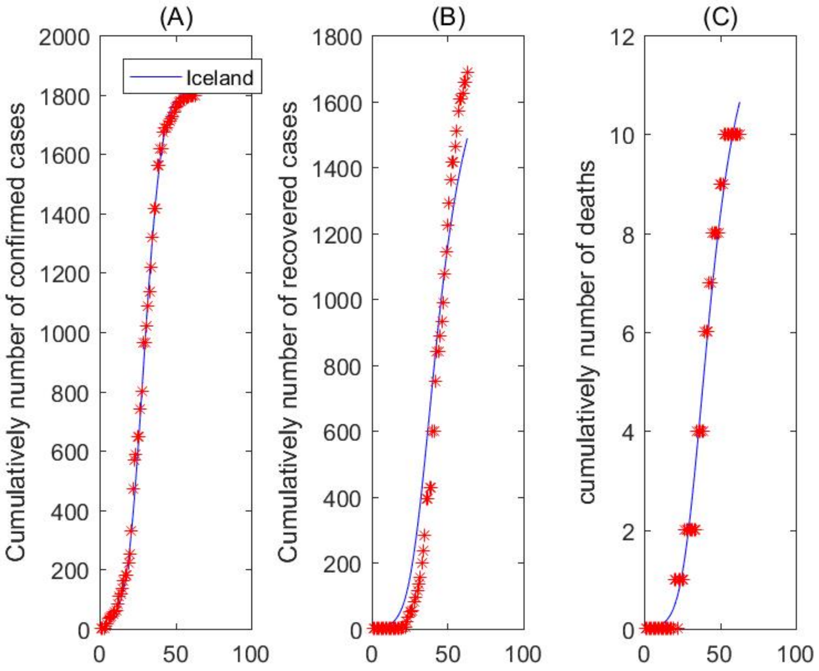

Figure S6: Simulation results of the cumulative confirmed cases, cumulative recovered cases, and cumulative deaths of Iceland in polar region.

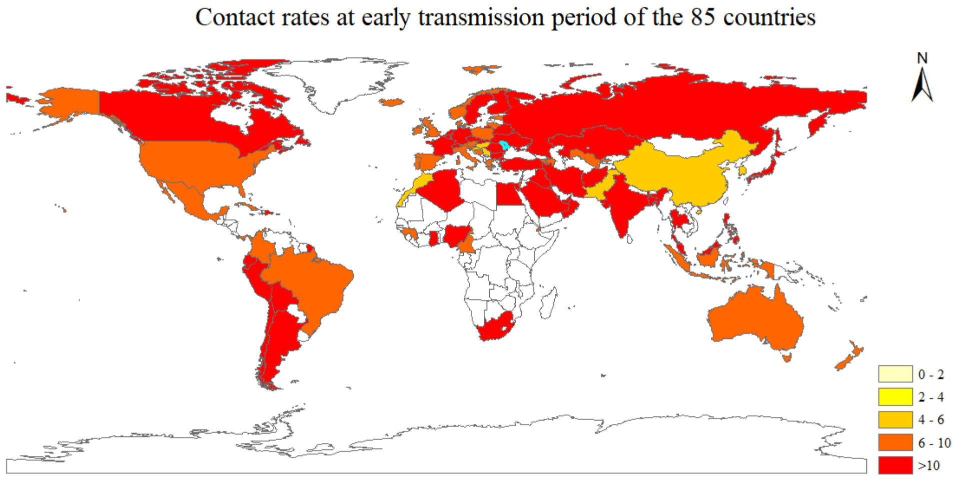

Figure S7: Distribution of the contact rates c_0 at early transmission period of the 85 countries.

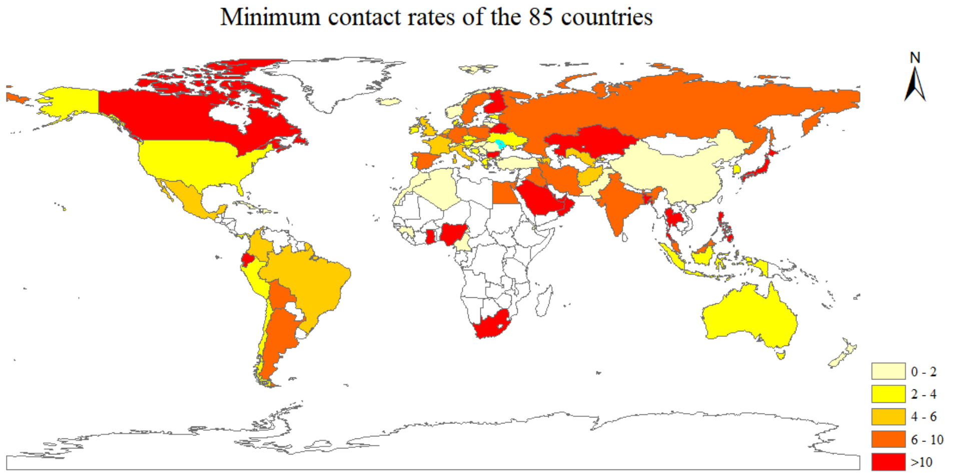

Figure S8: Same as Figure S5, but for the minimum contact rates c_f.

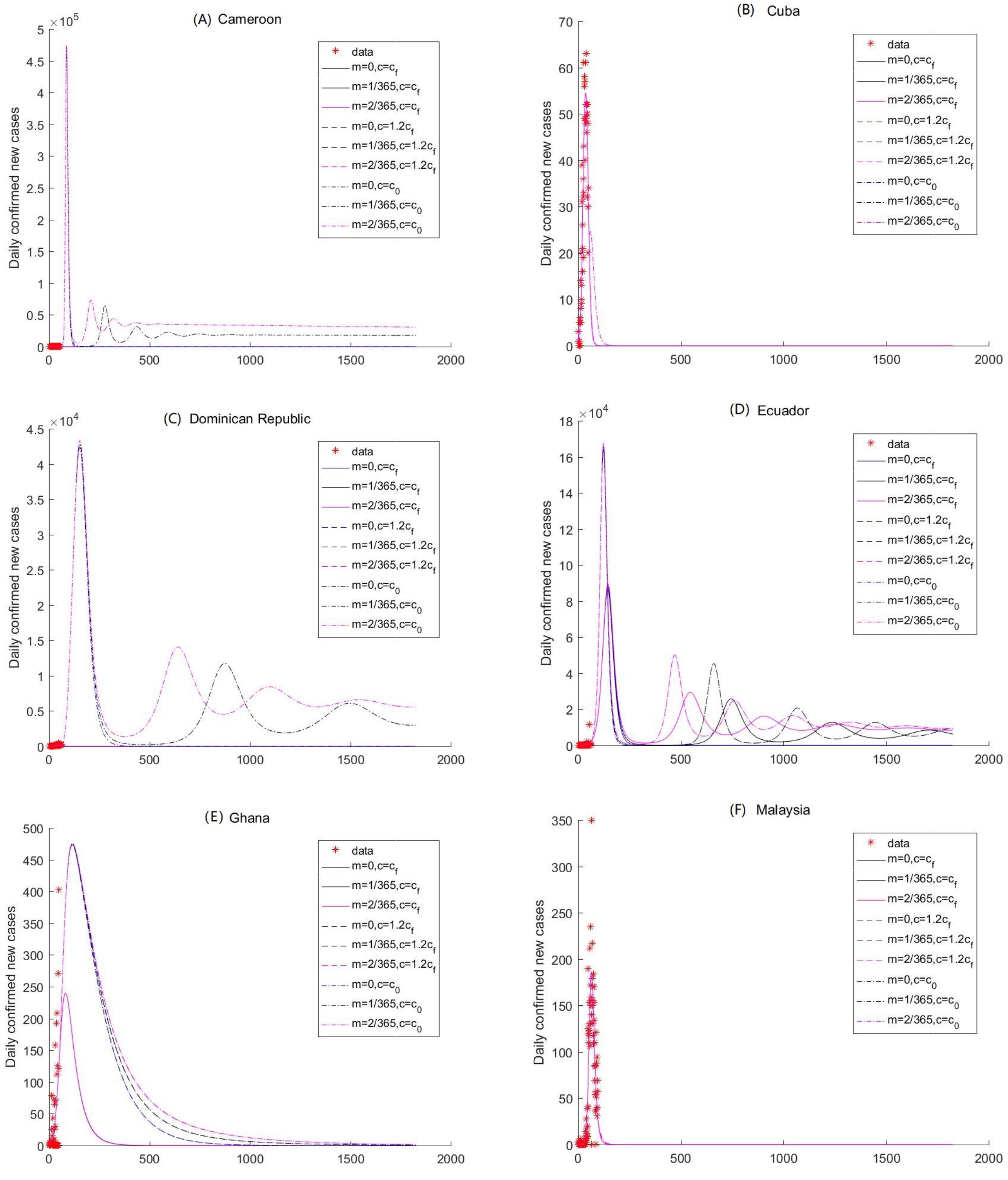
(A) Cameroon
×10^5
Daily confirmed new cases
data
m=0,c=c_f
m=1/365,c=c_f
m=2/365,c=c_f
m=0,c=1.2c_f
m=1/365,c=1.2c_f
m=2/365,c=1.2c_f
m=0,c=c_0
m=1/365,c=c_0
m=2/365,c=c_0
(B) Cuba
Daily confirmed new cases
data
m=0,c=c_f
m=1/365,c=c_f
m=2/365,c=c_f
m=0,c=1.2c_f
m=1/365,c=1.2c_f
m=2/365,c=1.2c_f
m=0,c=c_0
m=1/365,c=c_0
m=2/365,c=c_0
(C) Dominican Republic
×10^4
Daily confirmed new cases
data
m=0,c=c_f
m=1/365,c=c_f
m=2/365,c=c_f
m=0,c=1.2c_f
m=1/365,c=1.2c_f
m=2/365,c=1.2c_f
m=0,c=c_0
m=1/365,c=c_0
m=2/365,c=c_0
(D) Ecuador
×10^4
Daily confirmed new cases
data
m=0,c=c_f
m=1/365,c=c_f
m=2/365,c=c_f
m=0,c=1.2c_f
m=1/365,c=1.2c_f
m=2/365,c=1.2c_f
m=0,c=c_0
m=1/365,c=c_0
m=2/365,c=c_0
(E) Ghana
Daily confirmed new cases
data
m=0,c=c_f
m=1/365,c=c_f
m=2/365,c=c_f
m=0,c=1.2c_f
m=1/365,c=1.2c_f
m=2/365,c=1.2c_f
m=0,c=c_0
m=1/365,c=c_0
m=2/365,c=c_0
(F) Malaysia
Daily confirmed new cases
data
m=0,c=c_f
m=1/365,c=c_f
m=2/365,c=c_f
m=0,c=1.2c_f
m=1/365,c=1.2c_f
m=2/365,c=1.2c_f
m=0,c=c_0
m=1/365,c=c_0
m=2/365,c=c_0

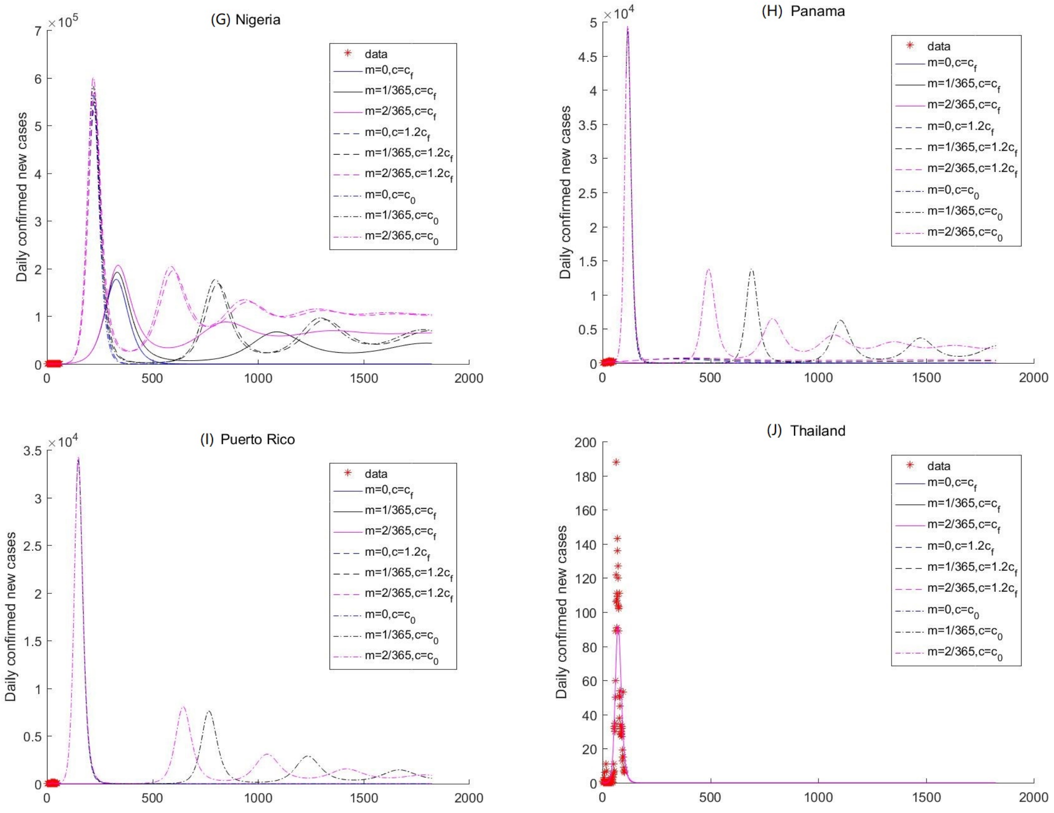

Figure S9: Sensitivity analysis of the daily new confirmed cases of Cameroon, Cuba, Dominican Republic, Ecuador, Ghana, Malaysia, Nigeria, Panama, Puerto Rico, and Thailand in tropical region.

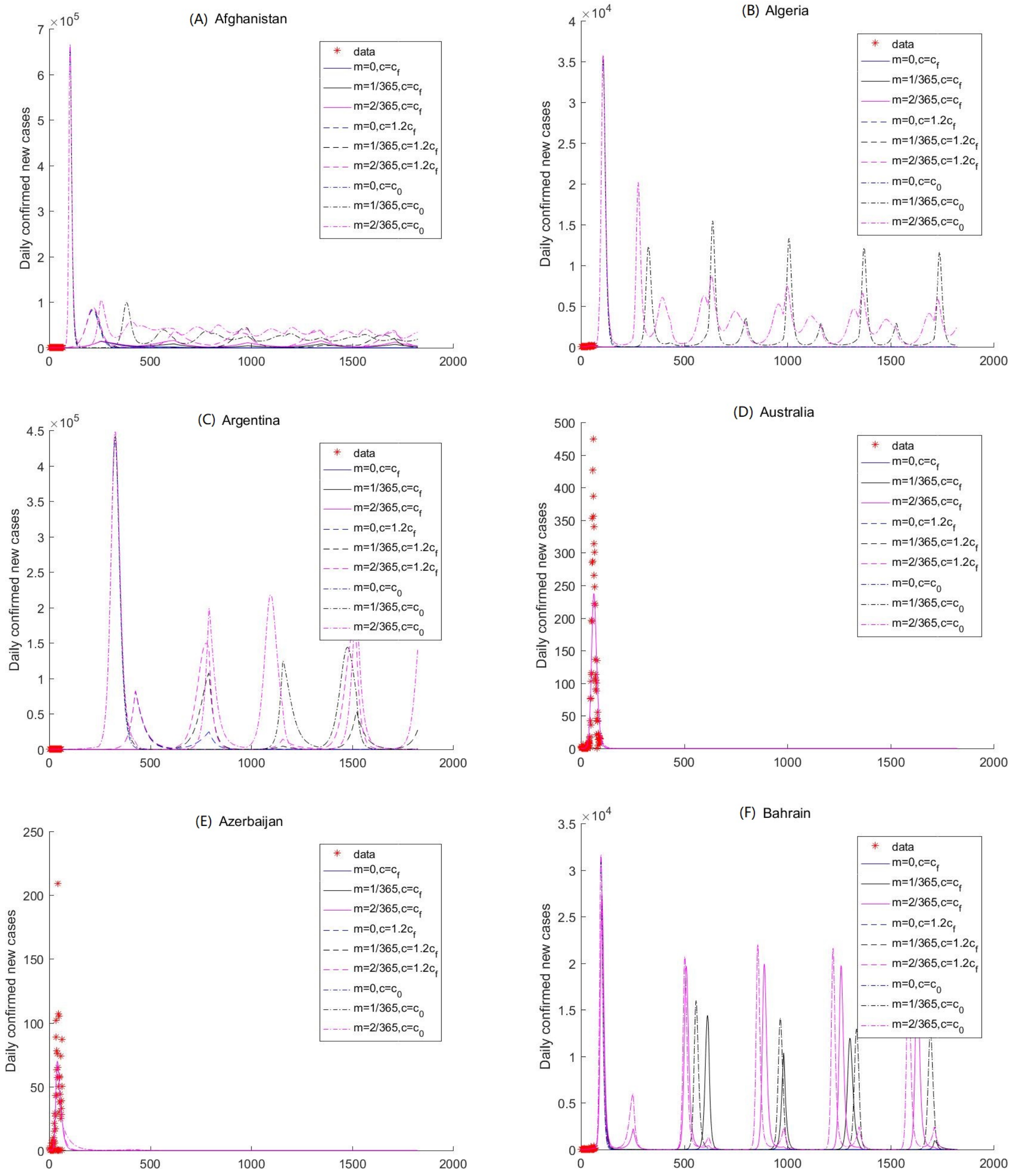
(A) Afghanistan
(B) Algeria
(C) Argentina
(D) Australia
(E) Azerbaijan
(F) Bahrain
Daily confirmed new cases
data
m=0,c=c_f
m=1/365,c=c_f
m=2/365,c=c_f
m=0,c=1.2c_f
m=1/365,c=1.2c_f
m=2/365,c=1.2c_f
m=0,c=c_0
m=1/365,c=c_0
m=2/365,c=c_0

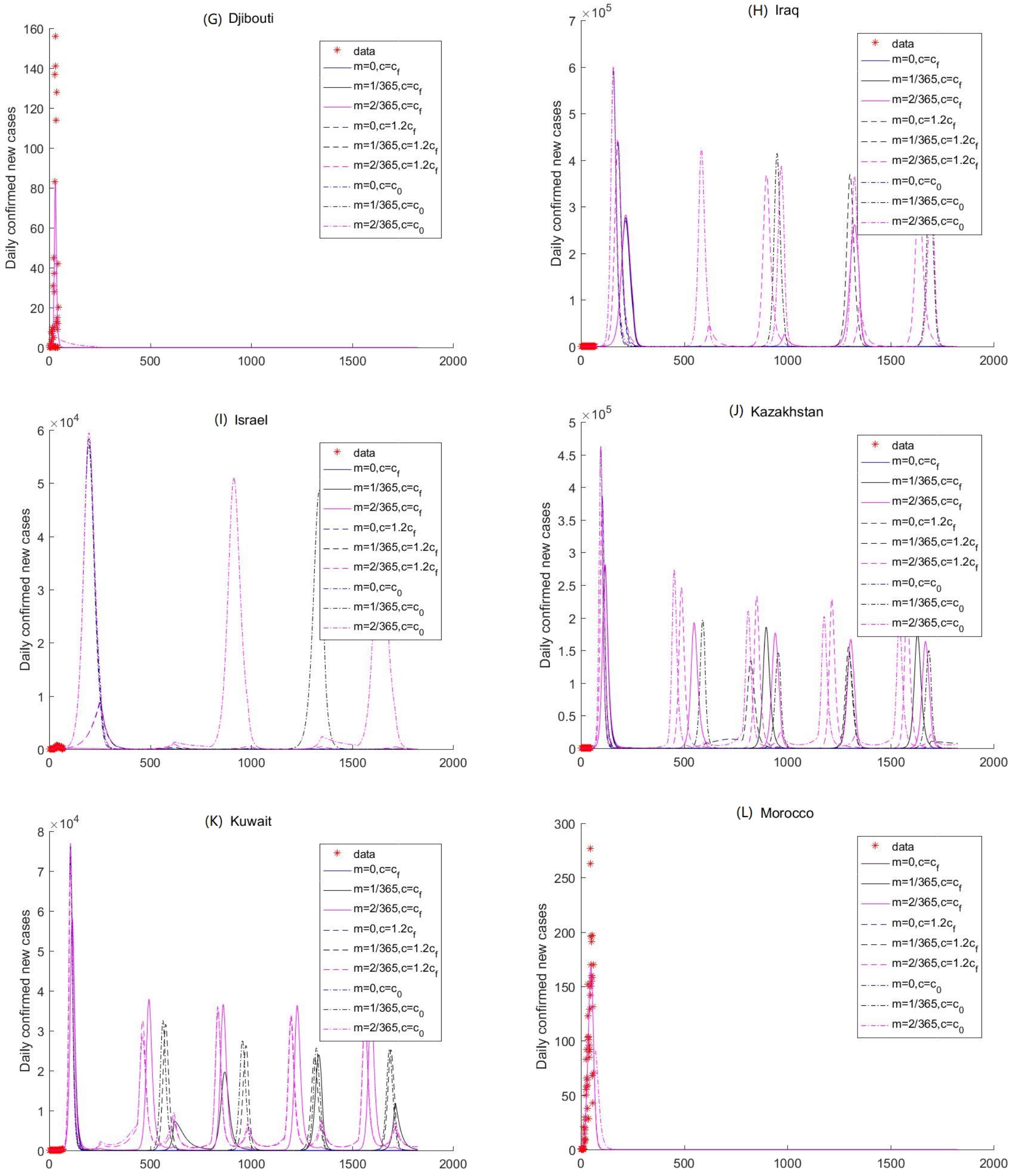

(G) Djibouti
(H) Iraq
(I) Israel
(J) Kazakhstan
(K) Kuwait
(L) Morocco
Daily confirmed new cases
data
m=0,c=c_f
m=1/365,c=c_f
m=2/365,c=c_f
m=0,c=$1.2c_f$
m=1/365,c=$1.2c_f$
m=2/365,c=$1.2c_f$
m=0,c=c_0
m=1/365,c=c_0
m=2/365,c=c_0

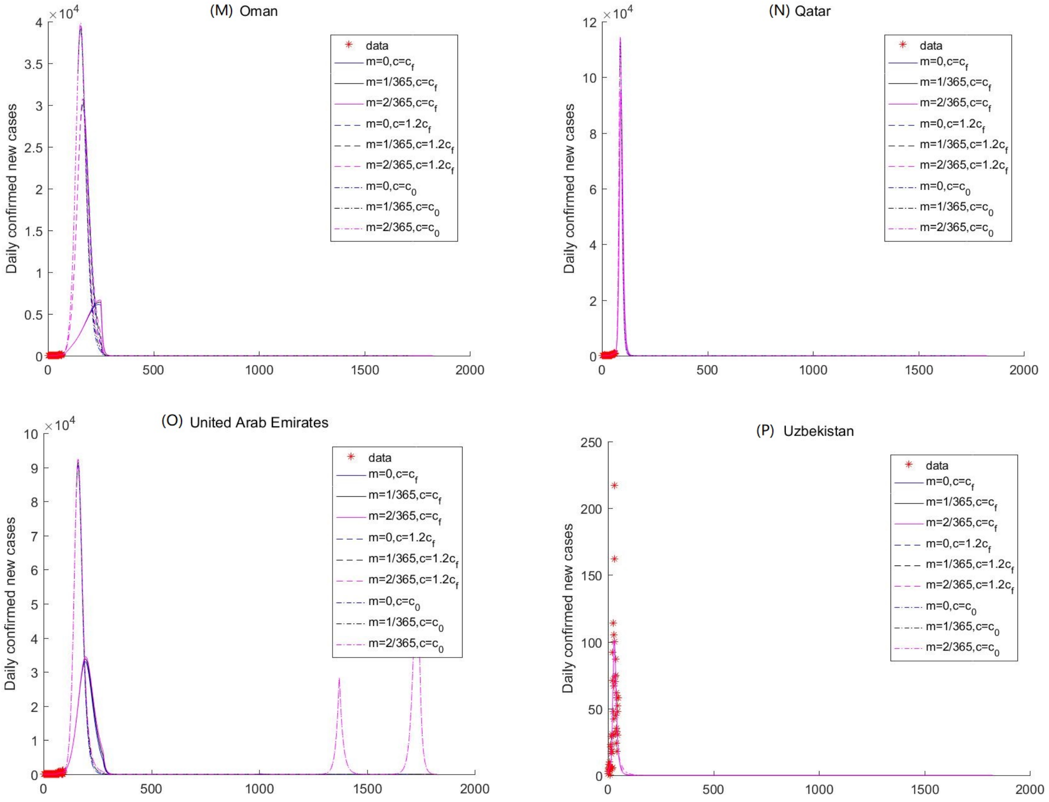

Figure S10: Sensitivity analysis of the daily new confirmed cases of Afghanistan, Algeria, Argentina, Austrialia, Azerbaijan, Bahrain, Djibouti, Iraq, Israel, Kazakstan, Kuwait, Morocco, Oman, Qatar, United Arab Emirates and Uzbekistan in arid region.

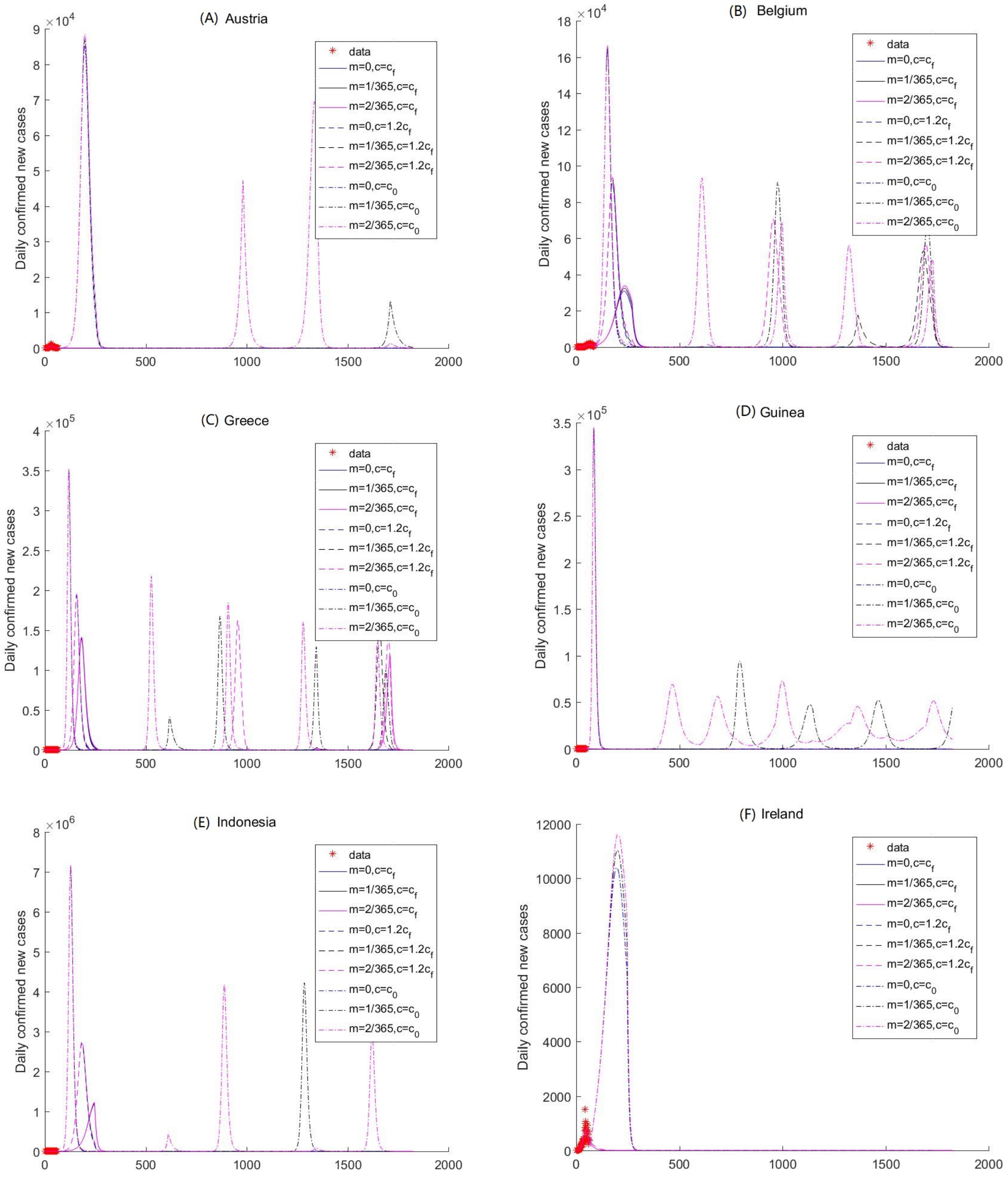
(A) Austria
(B) Belgium
(C) Greece
(D) Guinea
(E) Indonesia
(F) Ireland
Daily confirmed new cases
×10^4
×10^5
×10^6
data
m=0,c=c_f
m=1/365,c=c_f
m=2/365,c=c_f
m=0,c=1.2c_f
m=1/365,c=1.2c_f
m=2/365,c=1.2c_f
m=0,c=c_0
m=1/365,c=c_0
m=2/365,c=c_0
0
500
1000
1500
2000

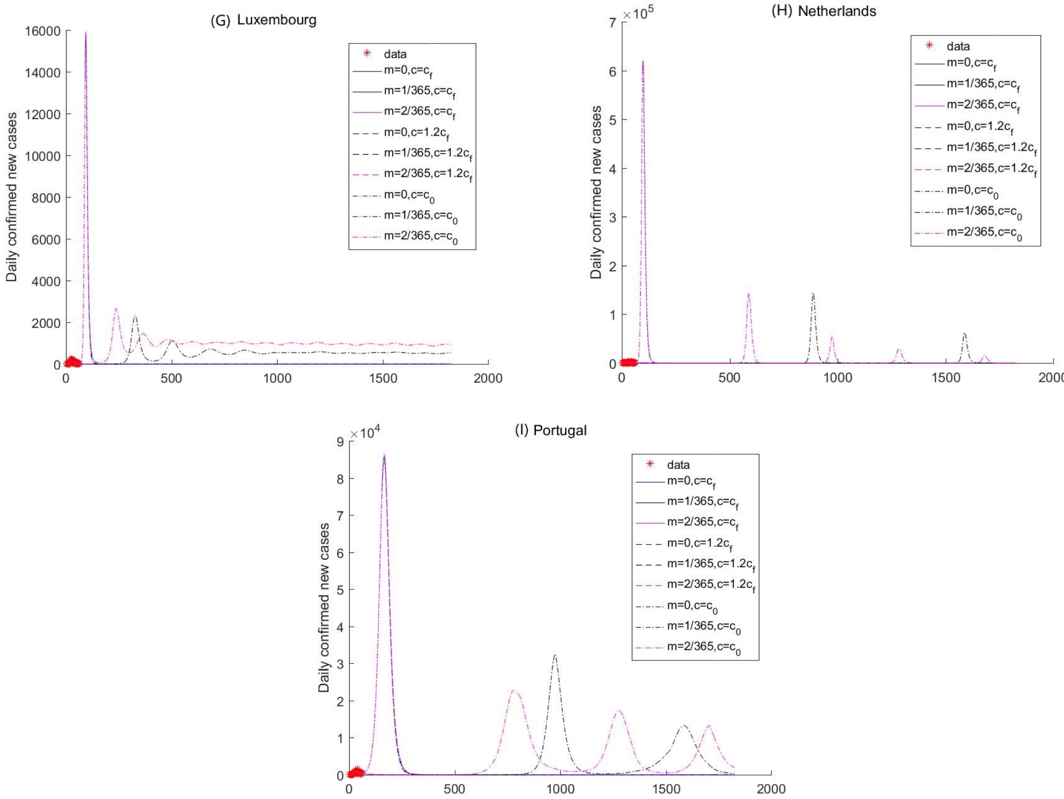

Figure S11: Sensitivity analysis of the daily new confirmed cases of Austria, Belgium, Greece, Guinea, Indonesia, Ireland, Luxembourg, Netherlands, and Portugal in temperate region.

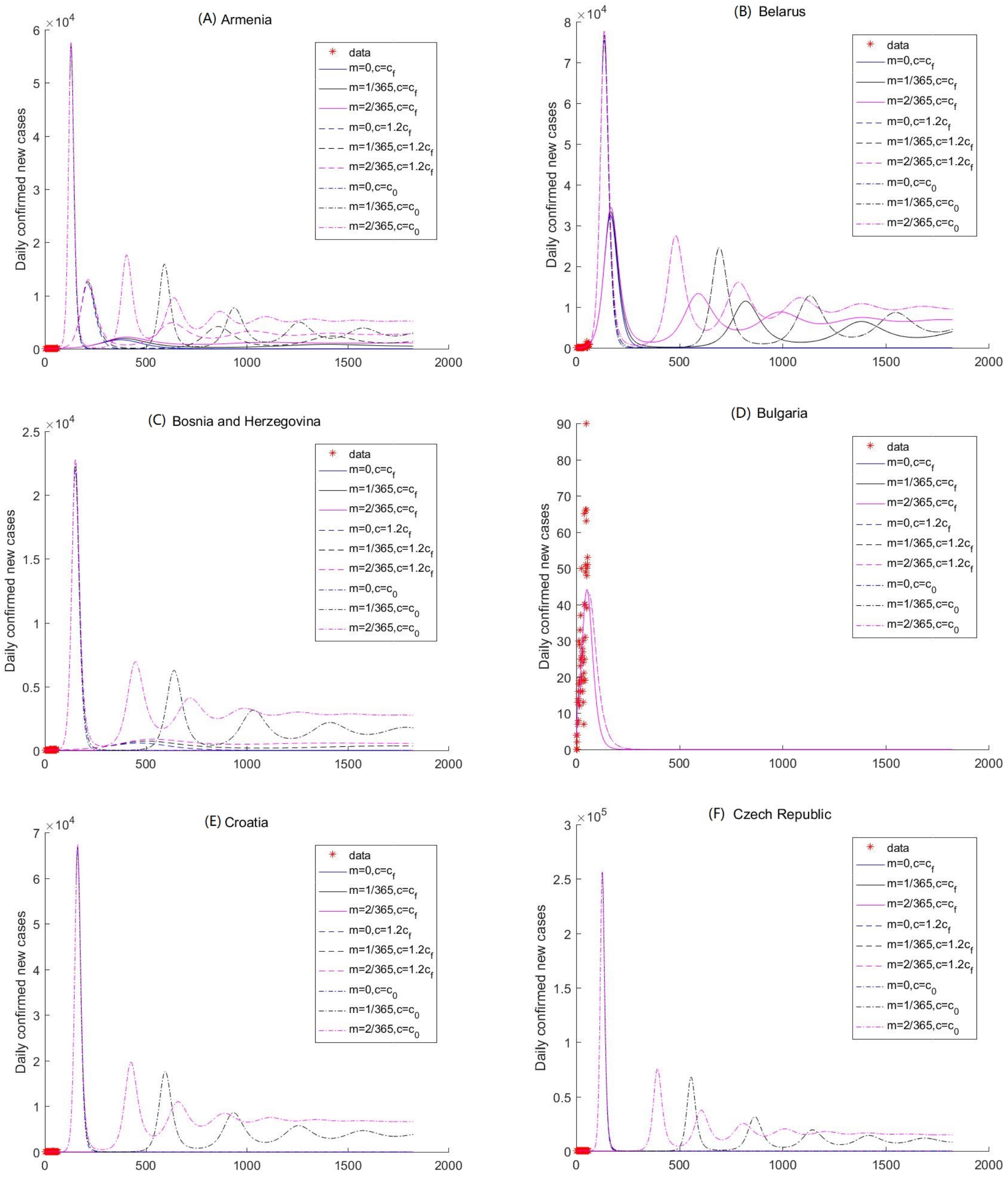
(A) Armenia
(B) Belarus
(C) Bosnia and Herzegovina
(D) Bulgaria
(E) Croatia
(F) Czech Republic
Daily confirmed new cases
data
m=0,c=c$_f$
m=1/365,c=c$_f$
m=2/365,c=c$_f$
m=0,c=1.2c$_f$
m=1/365,c=1.2c$_f$
m=2/365,c=1.2c$_f$
m=0,c=c$_0$
m=1/365,c=c$_0$
m=2/365,c=c$_0$

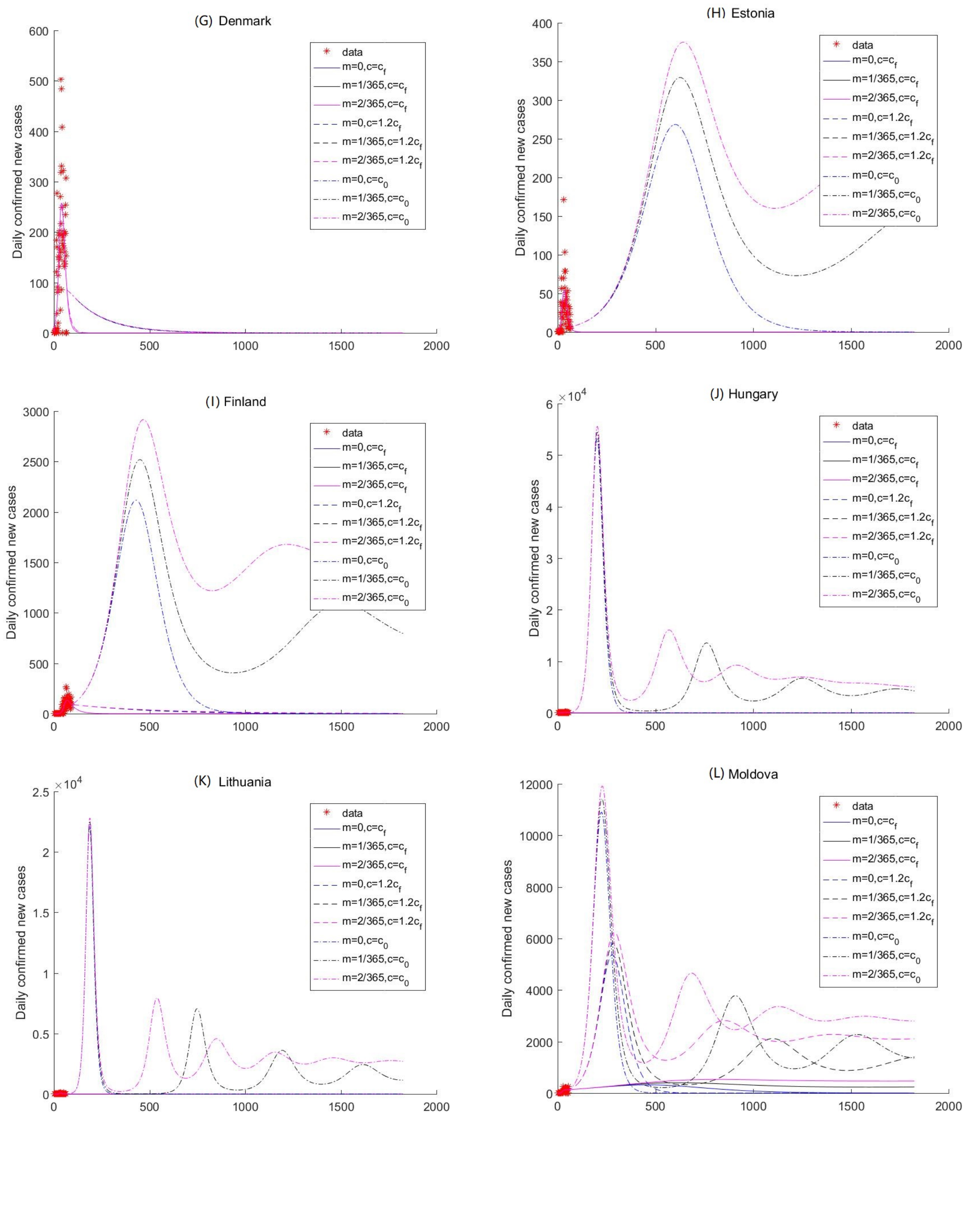
(G) Denmark
Daily confirmed new cases
data
m=0,c=c$_f$
m=1/365,c=c$_f$
m=2/365,c=c$_f$
m=0,c=1.2c$_f$
m=1/365,c=1.2c$_f$
m=2/365,c=1.2c$_f$
m=0,c=c$_0$
m=1/365,c=c$_0$
m=2/365,c=c$_0$
(H) Estonia
Daily confirmed new cases
data
m=0,c=c$_f$
m=1/365,c=c$_f$
m=2/365,c=c$_f$
m=0,c=1.2c$_f$
m=1/365,c=1.2c$_f$
m=2/365,c=1.2c$_f$
m=0,c=c$_0$
m=1/365,c=c$_0$
m=2/365,c=c$_0$
(I) Finland
Daily confirmed new cases
data
m=0,c=c$_f$
m=1/365,c=c$_f$
m=2/365,c=c$_f$
m=0,c=1.2c$_f$
m=1/365,c=1.2c$_f$
m=2/365,c=1.2c$_f$
m=0,c=c$_0$
m=1/365,c=c$_0$
m=2/365,c=c$_0$
(J) Hungary
$\times 10^4$
Daily confirmed new cases
data
m=0,c=c$_f$
m=1/365,c=c$_f$
m=2/365,c=c$_f$
m=0,c=1.2c$_f$
m=1/365,c=1.2c$_f$
m=2/365,c=1.2c$_f$
m=0,c=c$_0$
m=1/365,c=c$_0$
m=2/365,c=c$_0$
(K) Lithuania
$\times 10^4$
Daily confirmed new cases
data
m=0,c=c$_f$
m=1/365,c=c$_f$
m=2/365,c=c$_f$
m=0,c=1.2c$_f$
m=1/365,c=1.2c$_f$
m=2/365,c=1.2c$_f$
m=0,c=c$_0$
m=1/365,c=c$_0$
m=2/365,c=c$_0$
(L) Moldova
Daily confirmed new cases
data
m=0,c=c$_f$
m=1/365,c=c$_f$
m=2/365,c=c$_f$
m=0,c=1.2c$_f$
m=1/365,c=1.2c$_f$
m=2/365,c=1.2c$_f$
m=0,c=c$_0$
m=1/365,c=c$_0$
m=2/365,c=c$_0$

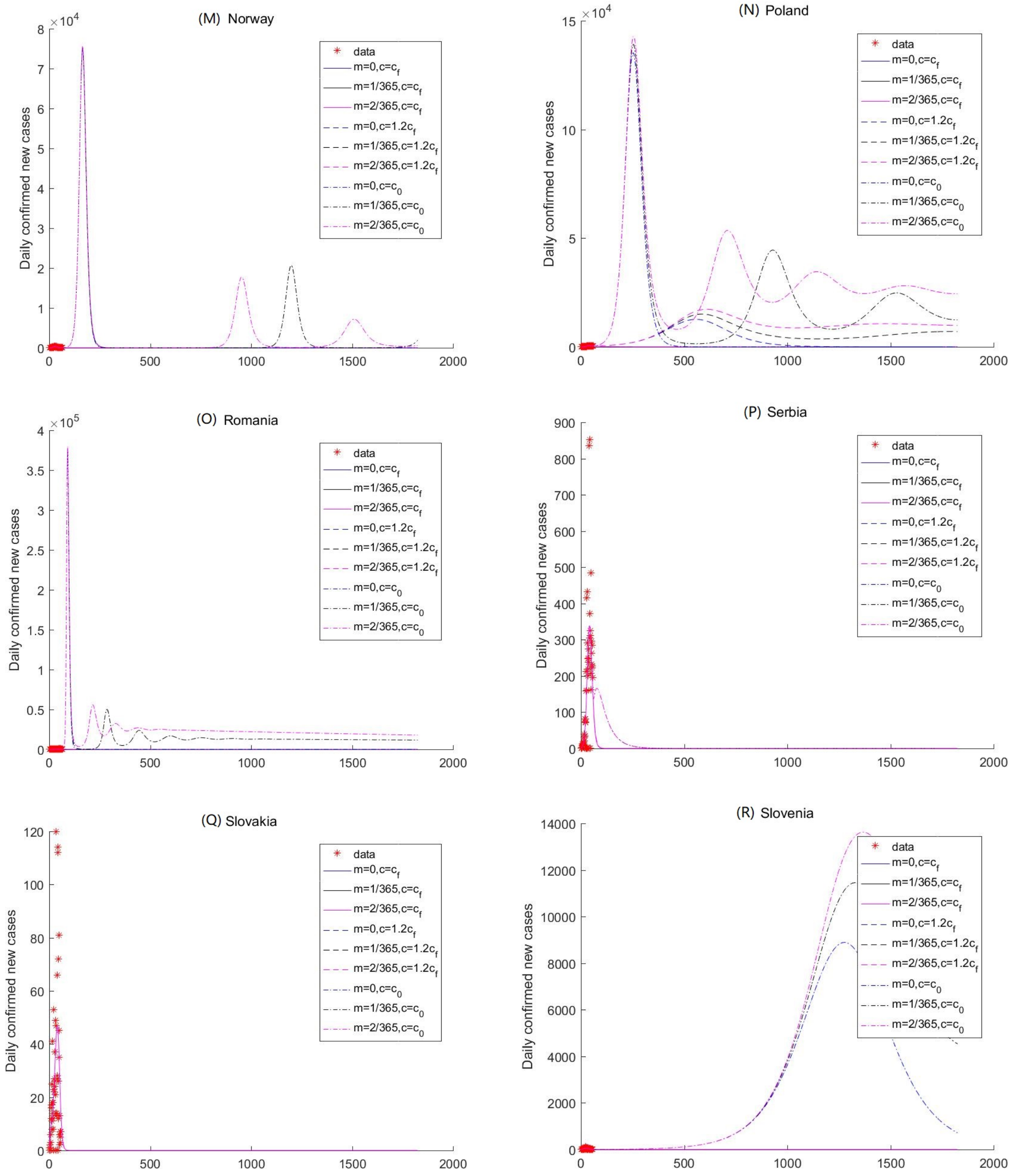
(M) Norway
(N) Poland
(O) Romania
(P) Serbia
(Q) Slovakia
(R) Slovenia
Daily confirmed new cases
×10^4
×10^5
data
m=0,c=c_f
m=1/365,c=c_f
m=2/365,c=c_f
m=0,c=1.2c_f
m=1/365,c=1.2c_f
m=2/365,c=1.2c_f
m=0,c=c_0
m=1/365,c=c_0
m=2/365,c=c_0

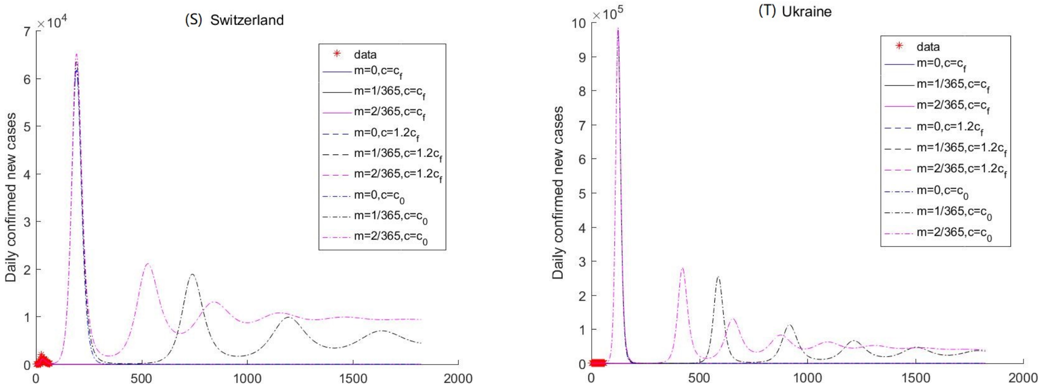

Figure S12: Sensitivity analysis of the daily new confirmed cases of Armenia, Belarus, Bosnia and Herzegovina, Bulgaria, Croatia, Czech Republic, Denmark, Estonia, Finland, Hungary, Lithuania, Moldova, Norway, Poland, Romania, Servia, Slovakia, Slovenia, Switzerland, and Ukraine in cold region.
