# Supplementary material for: Dynamical Variations of the Global COVID‐19 Pandemic Based on a SEICR Disease Model: A New Approach of Yi Hua Jie Mu
Source: Geohealth. 2021 Aug 1;5(8):e2021GH000455. doi: 10.1029/2021GH000455 (PMC8381858; doi:10.1029/2021GH000455)
Supplement: Supplementary file 2 — Table S1 [file GH2-5-e2021GH000455-s002.pdf]

Table Captions

Table S1 84 countries in the five climate regions: tropical climate, arid climate, temperate climate, cold climate and polar climate base on the Koppen-Geiger climate classification criteria.

Table S2 12 estimated parameters and 3 statistical metrics (CC: correlation coefficient, AE: absolute error, DISO: distance between indices of simulation and observation) of the 84 countries. Where N, S represent the northern hemisphere and southern hemisphere; A, B, C, D, E represent the tropical climate, arid climate, temperate climate, cold climate and polar climate.

Table S1 85 countries in the five climate regions: tropical climate, arid climate, temperate climate, cold climate and polar climate base on the Koppen-Geiger climate classification criteria.

Climate regions	Countries					
Tropical climate	Bolivia	Brazil	Cameroon	Colombia	Cuba	Dominican Republic
	Ecuador	Ghana	India	Malaysia	Peru	Nigeria
	Panama	Philippines	Puerto Rico	Singapore	Thailand	
Arid climate	Afghanistan	Algeria	Argentina	Australia	Azerbaijan	Bahrain
	Chile	China	Djibouti	Egypt	Iran	Iraq
	Israel	Mexico	Kazakhstan	Kuwait	Morocco	Oman
	Qatar	Saudi Arabia	South Africa	Spain	Turkey	United Arab Emirates
	Uzbekistan					
Temperate climate	Austria	Bangladesh	Belgium	France	Germany	Greece
	Guinea	Indonesia	Ireland	Italy	Japan	Luxembourg
	Netherlands	New Zealand	Portugal	United Kingdom	Netherlands	New Zealand
Cold climate	Armenia	Belarus	Bosnia and Herzegovina	Bulgaria	Canada	Croatia
	Czech Republic	Denmark	Estonia	Finland	Hungary	Lithuania
	Moldova	Norway	Poland	Romania	Russia	Serbia
	Slovakia	Slovenia	South Korea	Sweden	Switzerland	Ukraine

Table S2 12 estimated parameters and 3 statistical metrics (CC: correlation coefficient, AE: absolute error, DISO: distance between indices of simulation and observation) of the 84 countries. Where N, S represent the northern hemisphere and southern hemisphere; A, B, C, D, E represent the tropical climate, arid climate, temperate climate, cold climate and polar climate.

	NA			NB	
	Cuba	India	Philippines	Afghanistan	Algeria
c0	6.473391	17.99993	17.99987	19.18607	11.83355
delta0	0.023963	0.01	0.01	0.01	0.01
alphaC	0.003706	0.003571	0.004335	0.002948	0.014827
gammaI	0.105591	0.177656	0.199994	0.199996	0.199997
gammaC	0.032575	0.024361	0.006633	0.011508	0.045085
cf	1.74794	14.99993	14.99983	4.803405	1.230857
rb	0.39233	0.399998	0.39996	0.268468	0.18541
deltaf	0.668788	0.71	0.670747	0.110002	0.110001
rd	0.081773	0.062332	0.09073	0.05	0.05
p	0.069742	0.036635	0.03927	0.066569	0.151795
R0	3.484791	3.514036	3.366098	6.081999	8.553778
Rf	0.318895	0.889606	0.710822	1.209116	0.714235
relative bias	0.122338	0.119231	0.692627	0.10073	-0.15757
Correlation coefficent	0.999822	0.99982	0.998544	0.998266	0.998802
DISO	0.999997	1.000434	1.001528	1.001473	1.000313

	NB				
	Azerbaijan	Bahrain	China	Djibouti	Egypt
c0	8.006466	17.99999	5.268128	9.368391	12.4351
delta0	0.010031	0.033129	0.01	0.01	0.021189
alphaC	0.001441	0.000373	0.002398	0.000261	0.006995
gammaI	0.188126	0.2	0.071429	0.071429	0.19998
gammaC	0.065094	0.049522	0.046451	0.049952	0.024218
cf	4.980231	14.99999	1.74408	0.100001	9.434349
rb	0.396904	0.399999	0.05	0.093453	0.399866
deltaf	0.546805	0.133129	0.71	0.71	0.121205
rd	0.200461	0.05	0.067832	0.249745	0.075996
p	0.096008	0.022816	0.139062	0.040303	0.038897
R0	3.879173	1.761656	8.996824	4.636904	2.186956
Rf	0.651998	1.104982	0.312708	0.035328	1.172342
relative bias	-0.27053	-0.11486	2.678121	-0.0136	1.361281
Correlation coefficent	0.999377	0.99596	0.993808	0.997008	0.999815
DISO	1.000388	1.000551	0.998805	1.001186	1.000124

	NB				
	Iran	Iraq	Israel	Kazakhstan	Kuwait
c0	11.48556	10.25557	9.496015	18.72308	14.99292
delta0	0.167351	0.010042	0.011043	0.199857	0.060944
alphaC	0.006966	0.005787	0.000891	0.000909	0.000633
gammaI	0.071432	0.148548	0.072022	0.19259	0.199982
gammaC	0.074694	0.069057	0.025218	0.024088	0.034893
cf	8.437349	7.245433	6.441313	13.63191	11.99284
rb	0.399979	0.398494	0.125846	0.399953	0.399958
deltaf	0.867345	0.18063	0.429571	0.305528	0.160945
rd	0.05714	0.159471	0.215372	0.050011	0.07029
p	0.05573	0.032269	0.053925	0.04376	0.042885
R0	2.680642	2.086739	6.164758	2.08774	2.464216
Rf	0.574508	0.711464	0.693061	1.262733	1.449582
relative bias	0.197539	-0.11186	-0.17356	0.060506	-0.24035
Correlation coefficent	0.999093	0.998241	0.999837	0.999291	0.998857
DISO	0.999977	1.000209	1.000029	1.00006	1.001363

	NB				
	Mexico	Morocco	Oman	Pakistan	Qatar
c0	9.952451	5.36876	14.56819	4.971046	16.45529
delta0	0.101969	0.01	0.010008	0.01	0.012526
alphaC	0.017626	0.003791	0.000467	0.002016	0.000105
gammaI	0.177966	0.071429	0.072039	0.199999	0.07151
gammaC	0.092334	0.013672	0.017411	0.02176	0.009826
cf	4.864495	1.591385	11.5562	1.573758	13.45325
rb	0.050027	0.4	0.396649	0.399993	0.399343
deltaf	0.210527	0.71	0.709219	0.110001	0.710101
rd	0.398634	0.083547	0.078516	0.05	0.050006
p	0.106124	0.069903	0.018064	0.185912	0.014681
R0	3.773003	4.608804	3.207461	4.400865	2.874792
Rf	1.448607	0.325895	0.524922	1.117705	1.045745
relative bias	-0.03705	0.472568	-0.09072	0.461512	0.239072
Correlation coefficent	0.999702	0.999244	0.999438	0.999115	0.999178
DISO	1.000374	1.000225	1.000483	1.000489	1.000853

	NB				
	Saudi Arabia	Spain	Turkey	United Arab Emirates	United States
c0	14.47283	9.463395	11.3827	17.99957	9.709275
delta0	0.016991	0.01	0.199988	0.01	0.01
alphaC	0.000881	0.008833	0.002233	0.000698	0.003983
gammaI	0.192148	0.071429	0.103888	0.19875	0.080743
gammaC	0.014987	0.036849	0.021394	0.018224	0.00822

cf	11.45962	6.005123	0.100141	14.99954	6.70927
rb	0.399847	0.072505	0.055116	0.399981	0.05
deltaf	0.716722	0.71	0.299996	0.709996	0.704733
rd	0.059099	0.14808	0.399976	0.062419	0.073671
p	0.031719	0.099154	0.094762	0.036069	0.074129
R0	2.194983	11.52337	3.549625	3.110051	7.931598
Rf	0.964744	0.765848	0.367956	0.831996	0.71783
relative bias	0.322516	0.191984	2.091825	-0.05728	1.155269
Correlation coefficent	0.999093	0.999589	0.999823	0.99909	0.999556
DISO	1.000792	1.000182	1.000008	1.001999	1.000787

	NB	NC			
	Uzbekistan	Austria	Bangladesh	Belgium	Brazil
c0	8.184808	7.340245	18.00005	10.06183	8.761158
delta0	0.043367	0.028074	0.01	0.01	0.010046
alphaC	0.000401	0.002567	0.003281	0.011714	0.01082
gammaI	0.153218	0.072188	0.199957	0.19918	0.137728
gammaC	0.035533	0.053602	0.002955	0.018327	0.072269
cf	5.18463	3.723683	14.99997	7.061719	4.789163
rb	0.061691	0.396964	0.099771	0.050001	0.311581
deltaf	0.743336	0.663903	0.533082	0.677825	0.262098
rd	0.179193	0.219949	0.185108	0.121085	0.301128
p	0.050319	0.082193	0.052614	0.093473	0.126294
R0	2.095041	6.017343	4.510695	4.496172	7.487622
Rf	0.324213	0.415882	1.105406	0.769077	1.512767
relative bias	-0.0399	0.113345	-0.34945	0.611867	-0.2205
Correlation coefficent	0.998905	0.999824	0.999791	0.999844	0.999518
DISO	1.000014	0.999969	1.000353	1.000111	1.000522

	NC				
	Cameroon	Colombia	Dominican Republic	Ecuador	France
c0	9.492254	9.942427	10.69862	17.99999	12.79555
delta0	0.01	0.092983	0.011912	0.2	0.01
alphaC	0.003343	0.003883	0.003811	0.003461	0.009272
gammaI	0.199996	0.130853	0.113308	0.2	0.071429
gammaC	0.04133	0.016915	0.01037	0.006984	0.018066
cf	0.9562	5.575623	1.477094	14.99999	4.673993
rb	0.399995	0.382788	0.393681	0.399999	0.05
deltaf	0.110023	0.197742	0.700477	0.3	0.71
rd	0.05	0.308576	0.071754	0.05	0.072445
p	0.199986	0.072646	0.094498	0.04898	0.06096
R0	9.039745	3.226837	8.073767	2.204122	9.57908
Rf	0.766594	1.232673	0.414111	1.510668	0.436846

relative bias	-0.12169	0.250526	0.57464	1.478985	0.780343
Correlation coefficent	0.991382	0.999287	0.999611	0.979518	0.996487
DISO	1.001216	1.00005	1.000108	1.016614	1.003933

	NC				
	Germany	Ghana	Greece	Guinea	Indonesia
c0	18.81672	17.99728	7.138195	8.770922	6.777624
delta0	0.01	0.015226	0.022894	0.010071	0.042376
alphaC	0.003444	0.000841	0.002706	0.000745	0.006817
gammaI	0.071429	0.071615	0.080468	0.198052	0.071481
gammaC	0.072925	0.009586	0.010581	0.026067	0.010164
cf	6.750704	14.99473	3.41983	1.540469	3.753583
rb	0.05	0.050044	0.386614	0.398491	0.053214
deltaf	0.71	0.11537	0.174496	0.607388	0.356422
rd	0.088863	0.050079	0.142307	0.05013	0.05001
p	0.044817	0.010122	0.035573	0.182876	0.044078
R0	10.35633	2.097721	2.456706	7.706946	2.623829
Rf	0.425942	1.251073	0.478668	1.117939	0.641197
relative bias	0.205364	0.198528	0.206366	0.005824	0.751881
Correlation coefficent	0.999738	0.996198	0.999207	0.998703	0.999776
DISO	1.000168	1.000903	0.999736	1.000927	1.000049

	NC				
	Ireland	Italy	Japan	Luxembourg	Malaysia
c0	6.128225	8.036487	18.00078	11.20151	12.5507
delta0	0.017721	0.01	0.01	0.055575	0.01
alphaC	0.00534	0.008387	0.002033	0.001339	0.001384
gammaI	0.071554	0.074965	0.071431	0.173354	0.094522
gammaC	0.040518	0.017404	0.013883	0.025799	0.047609
cf	3.109068	4.436377	14.99857	0.835687	9.550633
rb	0.050172	0.399963	0.398578	0.399993	0.050002
deltaf	0.717106	0.453987	0.709996	0.155582	0.709997
rd	0.084532	0.163169	0.057109	0.097667	0.086095
p	0.043469	0.0956	0.022466	0.148512	0.038812
R0	2.983888	9.042397	4.96613	7.266669	4.66044
Rf	0.305475	0.802095	0.624021	0.380534	0.489854
relative bias	0.721503	0.467122	-0.38225	0.24839	-0.10463
Correlation coefficent	0.999149	0.999944	0.998472	0.99965	0.99963
DISO	1.000315	1.000007	1.002438	0.999872	1.000169

	NC				
	Netherlands	Nigeria	Panama	Portugal	Puerto Rico
c0	16.18329	16.86809	7.456741	6.046566	18.71613
delta0	0.078154	0.010001	0.155537	0.126875	0.174435
alphaC	0.006923	0.003747	0.001946	0.002144	0.003704
gammaI	0.199996	0.199944	0.197588	0.088246	0.199994
gammaC	0.001	0.024218	0.003841	0.002673	0.001
cf	4.656963	13.86596	3.325681	3.038055	3.814382
rb	0.330706	0.399541	0.294889	0.074671	0.399988
deltaf	0.778153	0.110047	0.25605	0.528075	0.874398
rd	0.05	0.050001	0.050653	0.242884	0.058711
p	0.072154	0.027737	0.119637	0.143682	0.086611
R0	4.198049	2.228556	2.526297	4.038582	4.329348
Rf	0.525921	1.484907	0.921922	0.73089	0.431355
relative bias	0.016501	0.789146	0.092852	0.290766	0.163482
Correlation coefficent	0.999856	0.996418	0.999579	0.999848	0.998105
DISO	0.999981	1.004192	0.999928	0.99998	0.999686

	NC			ND	
	Singapore	Thailand	United Kingdom	Armenia	Belarus
c0	18	18	7.174881	9.349559	17.99991
delta0	0.01	0.01	0.01	0.192244	0.01
alphaC	0.000107	0.001535	0.01029	0.001331	0.000852
gammaI	0.2	0.071429	0.071429	0.085355	0.199858
gammaC	0.008325	0.065163	0.001	0.034402	0.018949
cf	15	15	4.174201	5.159436	14.99986
rb	0.4	0.4	0.064296	0.398786	0.399936
deltaf	0.229108	0.71	0.556076	0.507335	0.382843
rd	0.05	0.070412	0.097263	0.399464	0.170297
p	0.034208	0.021002	0.104852	0.126457	0.051902
R0	2.932092	4.64246	9.238751	4.259079	4.451748
Rf	1.385719	0.458404	0.736565	1.100821	1.343821
relative bias	-0.42694	-0.0288	-0.1548	0.603834	-0.2567
Correlation coefficent	0.993065	0.994608	0.999762	0.997592	0.999299
DISO	1.019029	1.003338	1.000251	0.999863	1.001087

	ND				
	Bosnia and Herzegovina	Bulgaria	Canada	Croatia	Czech Republic
c0	6.478302	17.99998	18	4.240905	10.50122
delta0	0.028147	0.019695	0.01	0.010016	0.179312
alphaC	0.003179	0.003044	0.004967	0.001845	0.00166
gammaI	0.192389	0.130459	0.2	0.071807	0.098007

gammaC	0.027956	0.010958	0.035085	0.032247	0.015252
cf	3.179862	14.99993	15	0.141038	2.039459
rb	0.389094	0.050002	0.4	0.050038	0.15286
deltaf	0.132607	0.119698	0.577441	0.178246	0.28241
rd	0.296674	0.05	0.084935	0.370415	0.088892
p	0.092303	0.010188	0.043834	0.136164	0.095031
R0	2.711418	1.221362	3.757167	7.057327	3.598522
Rf	0.903123	0.779341	0.888414	0.242291	0.514987
relative bias	0.019559	0.188882	0.221225	-0.19582	0.108214
Correlation coefficent	0.999382	0.995255	0.999497	0.999697	0.99977
DISO	0.999925	0.9985	1.000785	1.000006	0.999929
			ND		
	Denmark	Estonia	Finland	Hungary	Lithuania
c0	6.107866	6.143929	14.34974	4.649406	6.149698
delta0	0.04799	0.052792	0.010004	0.019767	0.017288
alphaC	0.004489	0.001348	0.002745	0.008434	0.001774
gammaI	0.072804	0.186411	0.198675	0.071833	0.17696
gammaC	0.054363	0.005906	0.03648	0.013828	0.016418
cf	3.098829	2.614569	10.83475	0.635325	1.517232
rb	0.052364	0.392527	0.063218	0.077137	0.050052
deltaf	0.213865	0.440529	0.426119	0.120369	0.344554
rd	0.084165	0.101748	0.099829	0.05005	0.399874
p	0.04477	0.108735	0.047487	0.067179	0.135536
R0	2.263758	2.792857	3.265411	3.409875	4.290936
Rf	0.537845	0.467721	0.837468	0.355754	0.497941
relative bias	1.066476	0.850237	0.552484	0.103281	0.103745
Correlation coefficent	0.998746	0.998811	0.999651	0.999339	0.998679
DISO	0.999835	0.99964	1.000235	1.000002	0.999841
			ND		
	Moldova	Norway	Poland	Romania	Russia
c0	12.05996	7.368357	9.682529	11.13287	10.54204
delta0	0.012808	0.178133	0.089174	0.010007	0.01
alphaC	0.00257	0.000977	0.003332	0.004484	0.001099
gammaI	0.073539	0.071429	0.071798	0.199817	0.190059
gammaC	0.02089	0.001	0.013673	0.021416	0.010975
cf	9.013071	1.864943	6.614747	0.955953	7.542038
rb	0.310429	0.145819	0.153558	0.182804	0.05
deltaf	0.117477	0.278133	0.326283	0.110057	0.709999
rd	0.275821	0.088392	0.144269	0.050004	0.061107
p	0.022077	0.090918	0.054082	0.173001	0.075265
R0	3.083456	2.684387	3.25308	9.179138	3.966088
Rf	1.041785	0.488644	0.902577	0.636146	1.023346

relative bias	0.149725	0.185195	0.271386	-0.18828	0.396332
Correlation coefficent	0.999242	0.999406	0.999903	0.999734	0.999544
DISO	1.000153	0.99973	1.000002	1.000069	1.001501
			ND		
	Serbia	Slovakia	Slovenia	South Korea	Sweden
c0	4.086788	10.14991	6.900642	5.793026	10.64219
delta0	0.052178	0.015447	0.08138	0.01	0.010024
alphaC	0.001632	0.000833	0.002568	0.000964	0.007685
gammaI	0.084019	0.071429	0.105732	0.071429	0.103098
gammaC	0.011131	0.017933	0.00684	0.031809	0.002888
cf	1.032082	7.149813	2.591537	2.739499	6.229471
rb	0.060297	0.050001	0.332845	0.086901	0.313593
deltaf	0.490176	0.715444	0.182539	0.709999	0.214989
rd	0.065146	0.085174	0.081802	0.1987	0.097353
p	0.121694	0.01	0.044453	0.14743	0.049685
R0	3.65161	1.168347	1.639397	10.48842	4.67417
Rf	0.386431	0.19421	0.408836	0.517182	0.986335
relative bias	0.246419	0.224956	0.088009	0.147601	2.210679
Correlation coefficent	0.999301	0.997907	0.999488	0.99728	0.999683
DISO	1.000138	0.999494	0.999703	0.999014	1.000216
	ND		NE	SA	
	Switzerland	Ukraine		Bolivia	Peru
c0	10.31769	10.22659		10.01187	12.59996
delta0	0.081772	0.010206		0.01	0.01
alphaC	0.004267	0.002476		0.00528	0.003876
gammaI	0.145388	0.073033		0.199984	0.199998
gammaC	0.052118	0.009228		0.007565	0.045943
cf	1.874143	2.441369		6.090962	2.201096
rb	0.050066	0.380431		0.399978	0.218712
deltaf	0.44988	0.218734		0.709337	0.110001
rd	0.255594	0.099513		0.050001	0.05
p	0.086883	0.075355		0.042801	0.136258
R0	3.946274	9.258054		2.040719	8.175553
Rf	0.364765	0.734191		1.006105	1.202916
relative bias	0.01713	0.499648		0.129968	-0.20721
Correlation coefficent	0.999809	0.999879		0.999038	0.998669
DISO	0.999969	1.00009		1.000201	1.001295
		SB		SC	
	Argentina	Australia	Chile	South Africa	New Zealand
c0	10.11513	6.483388	17.05675	16.59293	7.523986
delta0	0.199939	0.01	0.010001	0.2	0.010097

alphaC	0.003851	0.000828	0.001486	0.0015	0.000765
gammaI	0.071534	0.071429	0.199987	0.2	0.17202
gammaC	0.021153	0.048676	0.051542	0.027286	0.055639
cf	7.111305	3.464129	2.168132	13.4288	0.101654
rb	0.399958	0.05	0.39998	0.399999	0.117325
deltaf	0.540229	0.71	0.11002	0.3	0.251505
rd	0.39999	0.084629	0.050001	0.05	0.217022
p	0.093931	0.080612	0.091104	0.041954	0.153375
R0	3.49989	6.418324	7.400119	1.740335	6.336556
Rf	1.09188	0.386945	0.783284	1.165253	0.045393
relative bias	0.045542	0.196499	-0.16595	1.048383	-0.06725
Correlation coefficent	0.999734	0.993513	0.999147	0.994234	0.999802
DISO	0.999986	1.003262	1.000228	0.999523	0.999976
